# Rac-dependent feedforward autoactivation of NOX2 leads to oxidative burst

**DOI:** 10.1016/j.jbc.2021.100982

**Published:** 2021-07-20

**Authors:** Hanh My Hoang, Hope Elizabeth Johnson, Jongyun Heo

**Affiliations:** Department of Chemistry and Biochemistry, The University of Texas at Arlington, Arlington, Texas, USA

**Keywords:** allostery, autoactivation, NOX2, NOX1, superoxide, Rac, redox, ClC-3, chloride channel-3, dHL60, differentiated HL60, DIDS, 4,4-diisothiocyanatostilbene-2,2′-disulfonic acid, GAP, GTPase-activating protein, GEF, guanine nucleotide exchange factor, NOX2, NADPH oxidase 2, H_2_O_2_, hydrogen peroxide, MAP, mitogen-activated protein, PMA, phorbol myristate acetate, SITS, 4-acetamide-4′-isothiocyanatostilbene-2,2′-disulfonic acid, SOD, superoxide dismutase

## Abstract

NADPH oxidase 2 (NOX2) produces the superoxide anion radical (O_2_^−^), which has functions in both cell signaling and immune defense. NOX2 is a multimeric-protein complex consisting of several protein subunits including the GTPase Rac. NOX2 uniquely facilitates an oxidative burst, which is described by initially slow O_2_^−^ production, which increases over time. The NOX2 oxidative burst is considered critical to immune defense because it enables expedited O_2_^−^ production in response to infections. However, the mechanism of the initiation and progression of this oxidative burst and its implications for regulation of NOX2 have not been clarified. In this study, we show that the NOX2 oxidative burst is a result of autoactivation of NOX2 coupled with the redox function of Rac. NOX2 autoactivation begins when active Rac triggers NOX2 activation and the subsequent production of O_2_^−^, which in turn activates redox-sensitive Rac. This activated Rac further activates NOX2, amplifying the feedforward cycle and resulting in a NOX2-mediated oxidative burst. Using mutagenesis-based kinetic and cell analyses, we show that enzymatic activation of Rac is exclusively responsible for production of the active Rac trigger that initiates NOX2 autoactivation, whereas redox-mediated Rac activation is the main driving force of NOX2 autoactivation and contributes to generation of ∼98% of the active NOX2 in cells. The results of this study provide insight into the regulation of NOX2 function, which could be used to develop therapeutics to control immune responses associated with dysregulated NOX2 oxidative bursts.

NADPH oxidase (NOX) is a plasma or phagosome membrane–bound enzyme. It produces superoxide anion radical (O_2_^−^), which often inactivates infections and serves as a redox signaling molecule (1, 2, 3). Several NOX isoforms, named numerically as NOX1, NOX2, NOX3, NOX4, and NOX5, have been reported (4, 5, 6). However, only NOX1 and NOX2 function with multiple phox subunits and with Rac (3, 7). The NOX2 phox subunits include cytochrome b558, p40, p47, and p67 (Fig. 1). Cytochrome b558, which is comprised of p22 and gp91, is unstable without p22 (8, 9). Of these phox subunits, p47 and p67 have been shown to be phosphorylated to function with cytochrome b558 (Fig. 1) (10, 11, 12, 13, 14). Rac, another NOX2 subunit, is a small GTPase that belongs to the Rho family of GTPases (15). All small GTPases, including the Rho family, function by cycling between the active GTP-bound and inactive GDP-bound states (16, 17). Active Rac is essential to NOX2 functioning (Fig. 1) (3, 7).

**Figure 1:**
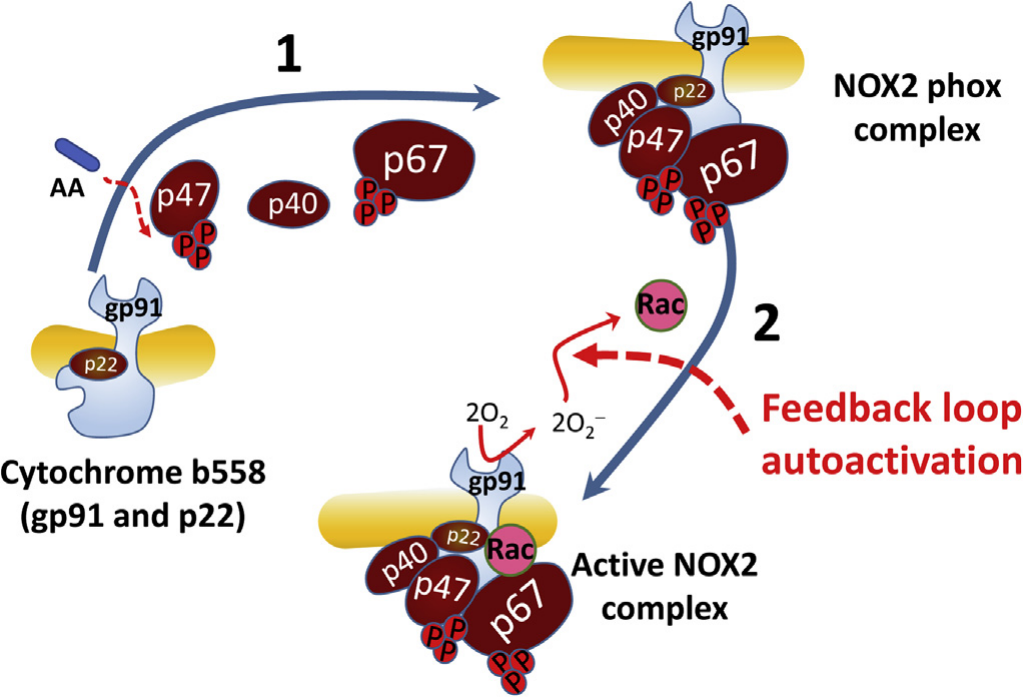
**Model of NOX2 assembly sequence.** The cytochrome b558 that consists of the membrane-anchored gp91 in complex with p22 is shown. Binding of p47 followed by p40 and p67 to the cytochrome b558 results in the formation of the NOX2 phox complex. Step 1: amphipathic arachidonic acid (AA) disables the autoinhibitory function of the “adaptor” protein p47 to make it bind to cytochrome b558 (18), which in turn recruits p40 and p67 onto cytochrome b558 to produce the NOX2 phox complex (19). Step 2: the binding of the active wt Rac to the NOX2 phox complex generates the O_2_^−^-producing active NOX2 complex. NOX2, NADPH oxidase 2.

A prospective assembly mechanism has been proposed for the formation of the active multimeric NOX2 enzyme complex (Fig. 1). Amphipathic arachidonic acid (or SDS *in vitro*) disables the autoinhibitory function of the “adaptor” protein p47, which allows it to bind to cytochrome b558 (18). In turn, this binding recruits p40 and p67 onto cytochrome b558 (step 1 in Fig. 1) (19). Ultimately, the binding of the active Rac (20, 21, 22) (step 2 in Fig. 1) to the NOX2 phox complex (13, 23, 24) completes the assembly of the O_2_^−^-producing NOX2 complex. The Rac effector region (Rac1 numbering 26–45) has been shown to be involved in the Rac-binding interaction with p67 on the NOX2 phox complex. Formation of this complex activates NOX2 (25, 26). Although a contradictory report exists (27), the Rac insert region (Rac1 numbering 124–135) has been reported as interacting with the NOX2 phox complex to activate the NOX2 function (22, 28, 29, 30). Interestingly, an active Rap1A also has been shown to aid the function of NOX2 but not of NOX1 (3). Rap1A belongs to the Ras family of small GTPases (15). However, the exact role of Rap1A in the NOX2 assembly process or in the NOX2 function is still debated (31). Thus, Rap1A was omitted from the depicted model.

Guanine nucleotide exchange factors (GEFs) and redox agents (32, 33, 34) can activate Rac. Studies have shown that Vav, a Rho GEF, activates Rac through a Theorell–Chance type of mechanism (16, 35, 36, 37). We have reported various redox agents relevant to the control of the small GTPase activities (34). Of them, O_2_^−^ is of interest because it is released from NOX2 (3). We have identified a distinct GXXXXGK(S/T)C motif that contains the redox-sensitive Cys18 (Rac1 numbering) conserved in many Rho proteins, including Rac (38, 39, 40). We have shown that O_2_^−^, but not its dismutation product hydrogen peroxide (H_2_O_2_), activates Rho proteins by targeting the GXXXXGK(S/T)C motif (34). In contrast to GEFs and redox agents, GTPase-activating proteins (GAPs) enhance the hydrolysis of the bound GTP to GDP, yielding an inactive GDP-bound Rac (33, 41, 42, 43).

The O_2_^−^ production kinetics between the NOX isoforms differ. The O_2_^−^ production of NOX2 as well as NOX1 is initially minimal but amplified over time (44, 45, 46). However, the O_2_^−^ production of NOX3, NOX4, and NOX5 is not amplified. The unique time-dependent amplification of the O_2_^−^ production by NOX1 and NOX2 has been known as an “oxidative burst” for decades. Previous studies have also suggested that this oxidative burst by NOX2 is an important immune cell response against infections because it enables NOX2 to expedite its O_2_^−^ production (44, 47, 48, 49). This NOX2 oxidative burst is kinetically described as hysteretic O_2_^−^ production over time. Time-dependent chemical or biochemical hysteresis can be generally described as a lagged or delayed reaction output that is not strictly a function of the corresponding input (50, 51, 52, 53). “Autoactivation” is one of the known time-dependent biochemical hysteretic events and described as an amplification of the positive feedback loop action of enzyme activation (53, 54, 55). Nevertheless, despite this operational knowledge, the mechanistic features of the hysteretic NOX2 oxidative burst over time have remained unknown.

We examined the kinetic features of the hysteretic NOX2 O_2_^−^ production over time. We found that the NOX2 oxidative burst is a reflection of the Rac redox-dependent NOX2 autoactivation. Our findings suggest a mechanism for NOX2 activation in which NOX2 is autoactivated through Rac-dependent amplification of the action of the positive feedback loop, which enables rapid activation of NOX2 in cells. Given that an active Rac also is required for NOX1 function and that NOX1 also displays an oxidative burst over time, discovery of the mechanism of NOX2 autoactivation is likely to be applicable to NOX1 as well.

## Results

We used cell-free and whole cell–based approaches to examine the mechanism of the NOX2 oxidative burst and its relevance to the regulation of NOX2 activity.

### NOX2 activation coupled with Rac activation

Studies show that the NOX2 oxidative burst is hysteretic and also time dependent (44, 45, 46). Notably, O_2_^−^ can be produced only after formation of the active NOX2 complex (Fig. 1). O_2_^−^ is one of the redox agents that elicits the redox response of wt Rac to produce active wt Rac (38). Accordingly, the observed hysteretic NOX2 activation may have occurred because of the feedback loop redox response of wt Rac to this NOX2-produced O_2_^−^. If so, this redox-dependent wt Rac activation could lead it to bind to the NOX2 phox complex to produce the active NOX2 complex. We have shown that the H_2_O_2_ dismutated from O_2_^−^ is unable to induce any redox response of wt Rac (38). However, studies also show that H_2_O_2_ is capable of oxidizing protein functional groups such as thiols (56, 57); therefore, this H_2_O_2_ may have an effect on the potential redox-mediated feedback loop between wt Rac and the NOX2 complex. To test for these possibilities, we examined the redox response of wt and redox-inert C18S Rac and its resultant active NOX2 complex formation under various redox conditions. Note that, although the exact mechanistic reason is unclear, C157S Rac also is redox inert. Therefore, as a control, C157S Rac also was used instead of C18S Rac in our examination in the cell-free and whole-cell systems.

#### Hysteretic redox-mediated activation of Rac and its function on NOX2 activation

**Figure 2:**
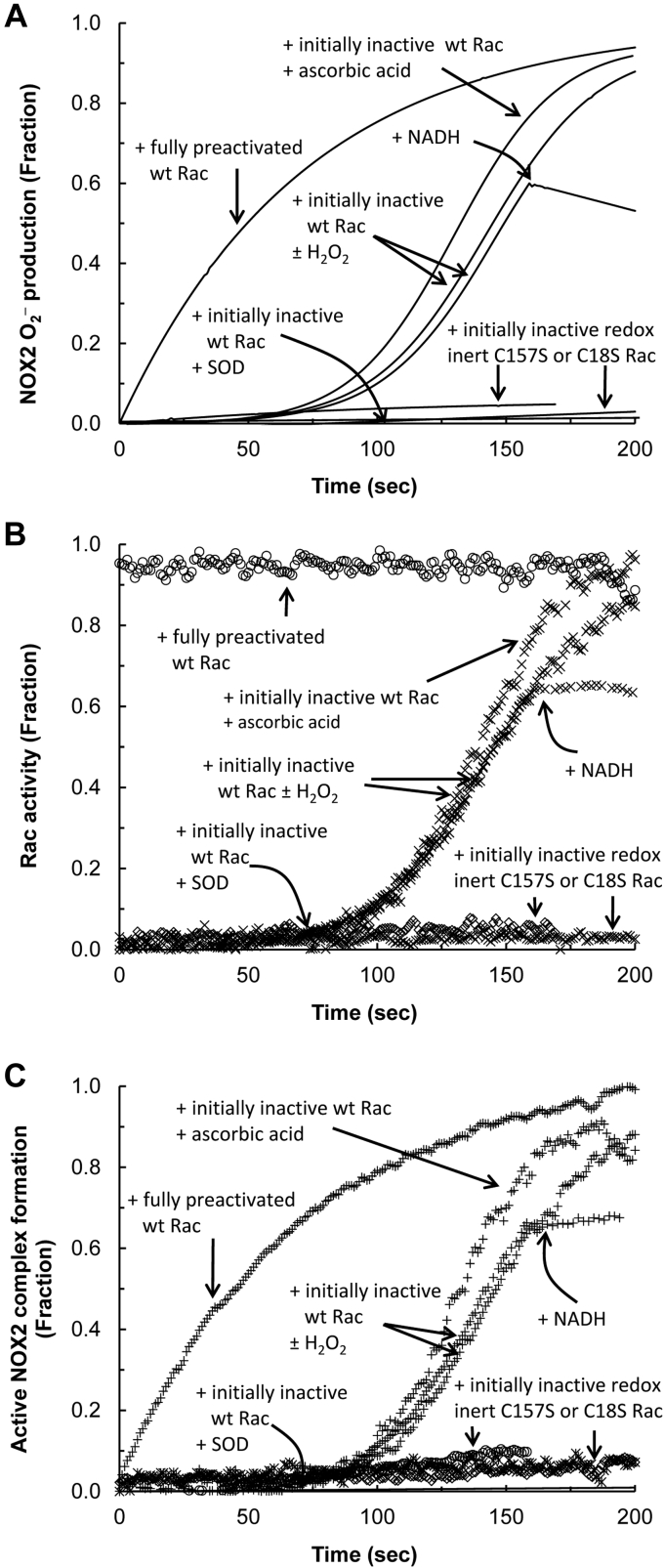
**Kinetics of the NOX2 O**_**2**_^**−**^**production and its dependency on the active NOX2 complex formation in the cell-free system.** NOX2 O_2_^−^ production and the NOX2 complex formation associated with wt Rac activation in the cell-free system were examined. All assays were initiated by the addition of fully preactivated or inactive wt Rac (100 μM) to the cell-free system that contained the NOX2 phox complex in the presence of SDS. The NOX2 phox complex is comprised of cytochrome b558 (gp91 plus p22), p40, p47, and p67 (Fig. 1). For comparisons, inactive C18S or C157S Rac also was used for this assay instead of inactive wt Rac. For all assays with inactive wt or mutant Rac, a fraction of preactivated wt Rac (∼2 mol %) was added prior to adding wt or mutant Rac as described in the Experimental procedures section. When necessary, SOD (500 units), H_2_O_2_ (5 w/w), NADH (10 mM), or ascorbic acid (10 mM) was added as indicated. *A*, NOX2 O_2_^−^ production using the cell-free assay system was measured by monitoring oxidation of cytochrome *c* over time as described in the Experimental procedures section. All NOX2 phox subunits used in this assay were unlabeled. *B*, the wt Rac activity was examined by monitoring the change in mant fluorescence intensity over time as described in the Experimental procedures section. Within this assay, mant-tagged GTP was used instead of the unlabeled GTP. All NOX2 phox subunits used in this assay were unlabeled. *C*, the active NOX2 complex formation was examined by monitoring the change in rhodamine fluorescence intensity over time as described in the Experimental procedures section. For this assay, the rhodamine-tagged cytochrome b558 was used instead of the unlabeled cytochrome b558. NOX2, NADPH oxidase 2; SOD, superoxide dismutase.

Figure 2*A* shows that the hysteretic O_2_^−^ production was linked only with the addition of inactive wt Rac to the cell-free NOX2 phox complex system containing a small fraction of preactivated wt Rac. It also is intriguing that, under identical experimental conditions, the inactive wt Rac that was added to the cell-free system of the NOX2 phox complex became activated hysteretically over time (Fig. 2*B*). Moreover, the kinetic profile of the binding of this hysteretically activated wt Rac to the NOX2 phox complex also was hysteretic over time (Fig. 2*C*). These results suggest that the hysteretic NOX2 O_2_^−^ production—equivalent to hysteretic NOX2 activation—is rooted in the kinetic profile of the hysteretic activation of the initially inactive wt Rac, followed by its binding to the NOX2 phox complex to produce the active NOX2 complex. It is noteworthy that the presence of a small fraction of preactivated wt Rac in the cell-free NOX2 phox complex system was essential for all the observed NOX2-relevant hysteretic processes over time. Without this small fraction of preactivated wt Rac in the cell-free assay system, no time-dependent hysteretic events were observed (not shown). The presence of this small fraction of preactivated wt Rac was determined to be one of the indispensable components required for the wt Rac-dependent NOX2 autoactivation (see the section on Rac-dependent autoactivation of NOX2 and its kinetic features).

The hysteretic production of NOX2 O_2_^−^ as well as the activation of wt Rac and its binding to the NOX2 phox complex over time become minimized when an excess of NADH is added to the cell-free system during activation of the hysteretic NOX2 (Fig. 2, *A*–*C*). NADPH—but not NADH—is a substrate of NOX2 to produce O_2_^−^. An excess of NADH competes with NADPH to minimize O_2_^−^ production from NOX2. Therefore, the results suggest that O_2_^−^ produced from NOX2 is involved in the hysteretic wt Rac and NOX2 activation over time. However, the hysteretic NOX2 O_2_^−^ production and its relevant processes of wt Rac activation and its binding to the NOX2 phox complex over time were unaffected by treatment with an excess of H_2_O_2_ (Fig. 2, *A*–*C*). Thus, the results suggest that H_2_O_2_ dismutated from O_2_^−^ has no effect on the hysteretic wt Rac and NOX2 activation over time. When combined, these results suggest that the hysteresis of the wt Rac and NOX2 activation over time is because of the O_2_^−^ produced from NOX2, but not because of its dismutated form, H_2_O_2_. This notion explains the counteraction of the superoxide dismutase (SOD) (58, 59) on the hysteretic events of NOX2 O_2_^−^ production as well as on wt Rac activation and its binding to the NOX2 phox complex over time (Fig. 2, *A*–*C*). O_2_^−^ dismutation by SOD minimizes hysteretic O_2_^−^-dependent wt Rac activation, thus blocking the binding of wt Rac to the NOX2 phox complex to produce active NOX2 (Fig. 2, *A*–*C*).

Figure 2*A* shows that after C18S or C157S Rac was added to the cell-free NOX2 phox complex system in the presence of a small fraction of preactivated wt Rac, production of NOX2 O_2_^−^ was minimal. Neither C18S nor C157S Rac was activated during the assay (Fig. 2*B*), and neither C18S nor C157S Rac was capable of binding to the NOX2 phox complex to produce the active NOX2 complex under the same experimental conditions (Fig. 2*C*). These failures associated with the redox-inert Rac (such as C18S and C157S Rac) in conjunction with the role of O_2_^−^ in the hysteresis of the wt Rac and NOX2 activation (see previous one) suggest a detailed mechanistic feature of the hysteretic wt Rac and NOX2 activation over time: the O_2_^−^-mediated wt Rac redox response is required to hysteretically activate the wt Rac that in turn enables wt Rac to bind to the NOX2 phox complex; these interactions then result in the production of O_2_^−^ by the active NOX2 complex in the cell-free system.

Intriguingly, all the observed hysteretic events—NOX2 O_2_^−^ production, wt Rac activation, and wt Rac binding to the NOX2 phox complex—were further enhanced by the addition of a radical quencher, ascorbic acid, to the cell-free system (Fig. 2, *A*–*C*). It was shown that the radical quencher enhances the O_2_^−^-mediated wt Rac activation (38). The results thus suggest that the effect of the radical quencher on wt Rac can be applicable to the wt Rac activation in the NOX2 system. The enhancement of NOX2 O_2_^−^ production by ascorbic acid over time also supports the notion that the hysteretic wt Rac activation is responsible for the wt Rac binding to the NOX2 phox complex and thus NOX2 O_2_^−^ production.

**Figure 3:**
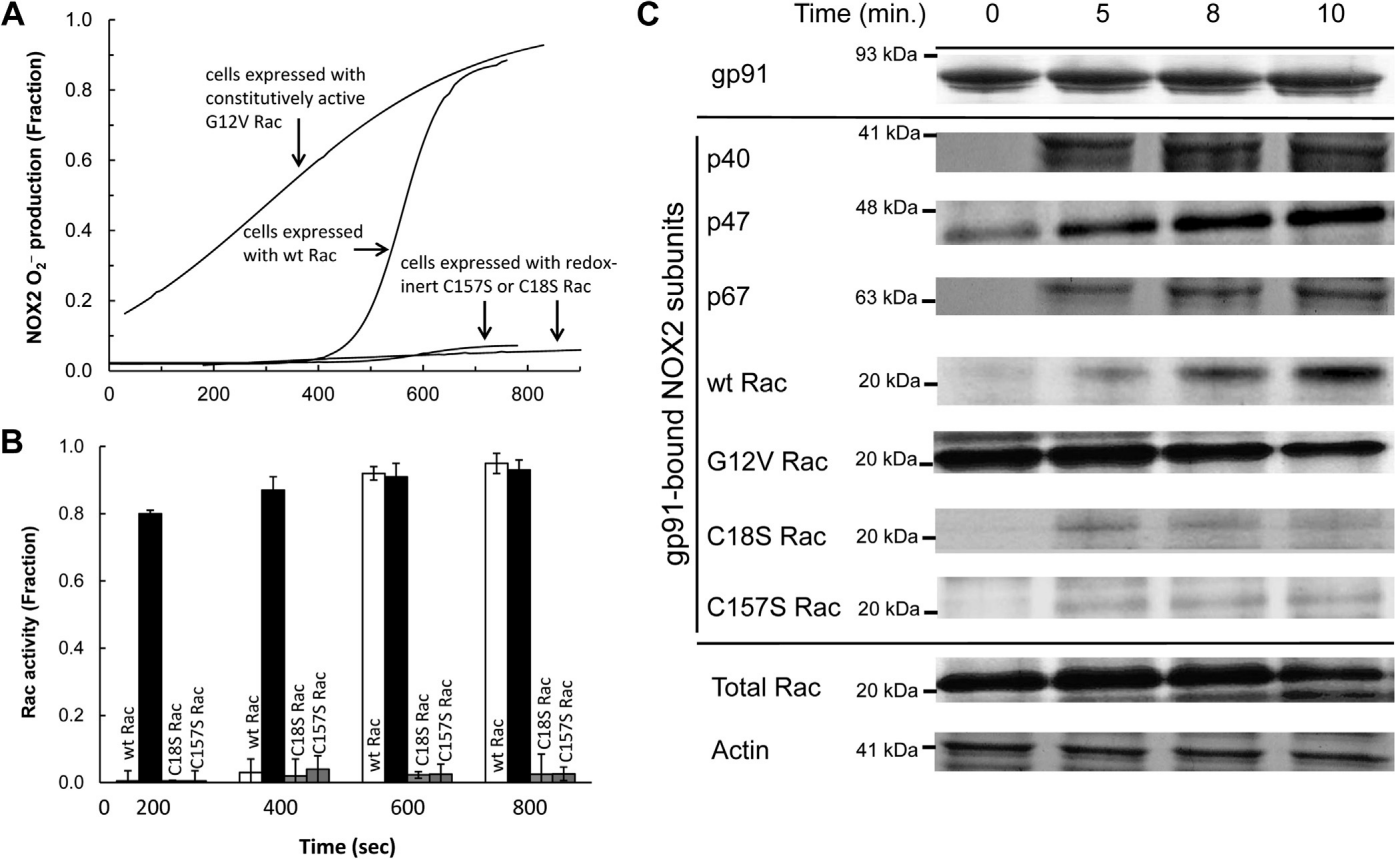
**NOX2 assembly and its link to the NOX2 O**_**2**_^**−**^**production in dHL60 cells.** Formation of the NOX2 complex associated with wt Rac activation and its resultant NOX2 O_2_^−^ production in cells was examined. For this analysis, dHL60 cells transfected with wt Rac, constitutively active G12V Rac, or redox inert C18S or C157S Rac were used. *A*, O_2_^−^ production from the PMA-stimulated dHL60 cells was examined by monitoring the oxidation of cytochrome *c* over time as described in the Experimental procedures section. *B*, the wt Rac activity change over time in the PMA-stimulated dHL60 cells was examined by using the colorimetric wt Rac activity assay kit (Abcam) as described in the Experimental procedures section. For this analysis, the pull-down NOX2 complexes resuspended in the sample buffer were used. All wt Rac activity assays were performed independently three times; their average values with SD are shown. *C*, the cellular active NOX2 complex formation in the PMA-stimulated dHL60 cells was examined by using pull-down and Western analyses as described in the Experimental procedures section. For this assay, as noted in the Experimental procedures section, the pull-down NOX2 complexes suspended in the sample buffer were used. *Upper panel*, the Western analysis of the pull-down sample with gp91 antibody is shown. *Middle panel*, the Western analyses of the pull-down sample with p40, p47, and p67 antibodies as well as a wt Rac antibody are shown. *Lower panel*, as loading controls, the cytosolic wt Rac and actin contents in the whole-cell lysates also were analyzed by using wt Rac and actin antibodies. NOX2, NADPH oxidase 2; PMA, phorbol myristate acetate.

Figure 3*A* shows that NOX2 O_2_^−^ production in the stimulated differentiated HL60 (dHL60) cells was hysteretic. This hysteretic NOX2 O_2_^−^ production over time was linked with the time-dependent hysteretic wt Rac activation (Fig. 3*B*) and with its recruitment to the NOX2 phox complex (Fig. 3*C*). Figure 3*A* also shows that, as with the results of the study of the cell-free system, NOX2 O_2_^−^ production was minimal from the stimulated dHL60 cells expressing C18S or C157S Rac. The pull-down assays in combination with the determination of Rac activity also show that the C18S or C157S Rac expressed in dHL60 cells was only minimally activated (Fig. 3*B*). C18S or C157S Rac expressed in dHL60 cells was minimally recruited to the NOX2 phox complex over time (Fig. 3*C*). All these cell study results also suggest that the O_2_^−^-mediated wt Rac redox response causes wt Rac activation coupled with the wt Rac recruitment to the NOX2 phox complex. In turn, this response also yields the active NOX2 that produces O_2_^−^.

#### Fully preactivated Rac and its function on NOX2 activation

Figure 2*A* shows that when the fully preactivated wt Rac instead of the inactive wt Rac was added to the same cell-free system, NOX2 O_2_^−^ production was only hyperbolic over time. The activity of the preactivated wt Rac also remained unchanged during the assay (Fig. 2*B*). The kinetic profile of the binding of the preactivated wt Rac to the NOX2 phox complex in the cell-free system was nevertheless hyperbolic rather than hysteretic over time (Fig. 2*C*). These results suggest that production of hyperbolic NOX2 O_2_^−^ occurs solely because of the kinetic profile of its hyperbolic binding of the fully preactivated wt Rac to the NOX2 phox complex to produce the active NOX2 complex.

The NOX2 O_2_^−^ production associated with the constitutively active G12V Rac and its recruitment to the NOX2 phox complex in the stimulated dHL60 cells were hyperbolic over time (Fig. 3, *A*–*C*). Moreover, upon stimulation of the dHL60 cells, the recruitment of the phox subunits to cytochrome b558 occurred earlier than the wt Rac recruitment to cytochrome b558 (Fig. 3*C*). This was also observed in the constitutively active G12V Rac-expressing dHL60 cells (not shown). The order in which cells in NOX2 are assembled is consistent with previous reports (31). This order of assembly in NOX2 also supports the notion that the final recruitment of active wt Rac is the key controlling factor that determines the kinetic mode of the formation of the active NOX2 complex, and thus NOX2 O_2_^−^ production. The results of the cell study are consistent with the cell-free study results in that the preactivated wt Rac elicits the hyperbolic production of O_2_^−^.

### Roles of Rac effector and insert regions in the hysteretic wt Rac and NOX2 activation

The cell-free and whole cell–based kinetic analyses suggest that the unique time-dependent hysteretic coupling between wt Rac and NOX2 activation is accompanied with the binding interaction of wt Rac with the NOX2 phox complex. It is unclear whether the time-dependent hysteresis of wt Rac and NOX2 activation is rooted in the kinetic process of wt Rac and NOX2 activation or governed by a previous unknown potential hysteretic-binding process of the Rac effector and/or insert region(s) to the NOX2 phox complex over time. To test these possibilities, we examined the kinetic profile of the NOX2 activation with the effector mutants A27K and G30S Rac (60) as well as the insert region mutants K132E and L134R Rac (28, 29) in the cell-free system. The experimental conditions for these examinations were identical to those with wt Rac in which a small fraction of preactivated wt Rac was present in the cell-free assay system.

Table 1 shows that no hysteretic NOX2 O_2_^−^ production over time occurred when we used the initially inactive A27K or G30S Rac instead of the initially inactive wt Rac (Table 1). Neither the initially inactive A27K nor the G30S Rac was activated, and both were incapable of binding to the NOX2 phox complex over time (Table 1). Neither the fully preactivated A27K nor G30S Rac was able to bind to the NOX2 phox complex over time (Table 1). The redox sensitivity of both A27K and G30S Rac was the same as that of wt Rac (Table 1). This lack of NOX2 activation regardless of the activity status of A27K or G30S Rac suggests that the interaction of the Rac effector region with p67 on the NOX2 phox complex is not responsible for the time-dependent hysteresis of wt Rac and NOX2 activation. Instead, it appears to function simply for the binding of the hysteretically activated Rac to p67 on the NOX2 phox complex.

**Table 1:** Role of the effector and insert regions of Rac in the hysteretic coupling of the Rac redox response and NOX2 activation

Rac		Hyperbolic NOX2 activation with fully preactivated Rac	Hysteretic Rac activation with initially inactive Rac	Hysteretic NOX2 activation with initially inactive Rac	Hyperbolic Rac activation with fully preactivated Rac	Rac redox sensitivity^a^
wt Rac		+++	+++	+++	+++	+++
Rac redox motif mutants	C18S	—	—	—	+++	—
	C157S	—	—	—	+++	—
Rac effector region mutants	A27K	—	—	—	—	+++
	G30S	—	—	—	—	+++
Rac insert region mutants	K132E	++	++	++	++	+++
	L134R	++	++	++	++	+++

a Rac redox sensitivity was measured without the NOX2 phox complex in the cell-free system by using KO_2_ as the O_2_^−^ source. The symbol “+++” represents the maximal wt Rac redox sensitivity or the fastest hysteretic activation rate of NOX2 and wt Rac over time based upon the results shown in Figure 2, *A* and *B*. The symbols “++” and “+” reflect, respectively, 30% and 60% slower rates than those of the rates shown as “+++.” The symbol “—” denotes a lack of the redox sensitivity or hysteretic activation over time.

Table 1 also shows that, unlike what happened with the Rac effector mutants, the hysteretic NOX2 O_2_^−^ production with the initially inactive K132E or L134R Rac was observed. However, the hysteretic process is ∼1.5-fold slower than that with the initially inactive wt Rac. The rate of the binding of the initially inactive K132E or L134R Rac to the NOX2 phox complex also was ∼1.5-fold slower than that of the initially inactive wt Rac to the NOX2 phox complex (Table 1). However, once the relatively slow hysteretic process associated with the initially inactive K132E or L134R Rac was completed, the final total activity of NOX2 was the same as that with wt Rac. Moreover, the binding of the fully preactivated K132E or L134R Rac to the NOX2 phox complex was hyperbolic over time; however, it also was 1.5-fold slower than that with the fully preactivated wt Rac. The redox sensitivity of K132E and L134R Rac was nevertheless nearly identical to that of wt Rac (Table 1). Given that both the hysteretic and hyperbolic processes associated with the initially inactive and fully preactivated K132E or L134R Rac, respectively, were consistently slower than those associated with wt Rac, the time-dependent hysteretic NOX2 activation feature is unlikely to be rooted in the Rac insert's region-binding interaction with the NOX2 phox complex. The relatively slow binding feature of active K132E or L134R Rac to the NOX2 phox complex may be because of the perturbation of the function of the insert region by the mutation (28, 29) or the potential instability of K132E and L134R Rac (27).

### Role of the O_2_^−^ channel in the hysteretic Rac redox response and NOX2 activation in cells

Unlike in the cell-free system, cells possess a plasma or phagocytic membrane that separates NOX2 subunits, including wt Rac, from the extracellular O_2_^−^ released by NOX2 (3). This notion raises the question, how does the extracellularly released O_2_^−^ cross the membrane to provide feedback to activate the wt Rac located within cells? Studies show that O_2_^−^ can cross these membranes through one of the chloride channels—chloride channel-3 (ClC-3)—to reach the place inside cells where wt Rac is located (61, 62, 63). Accordingly, ClC-3 on the plasma membrane may function as a conduit for O_2_^−^ that enables the wt Rac redox response in cells.

**Figure 4:**
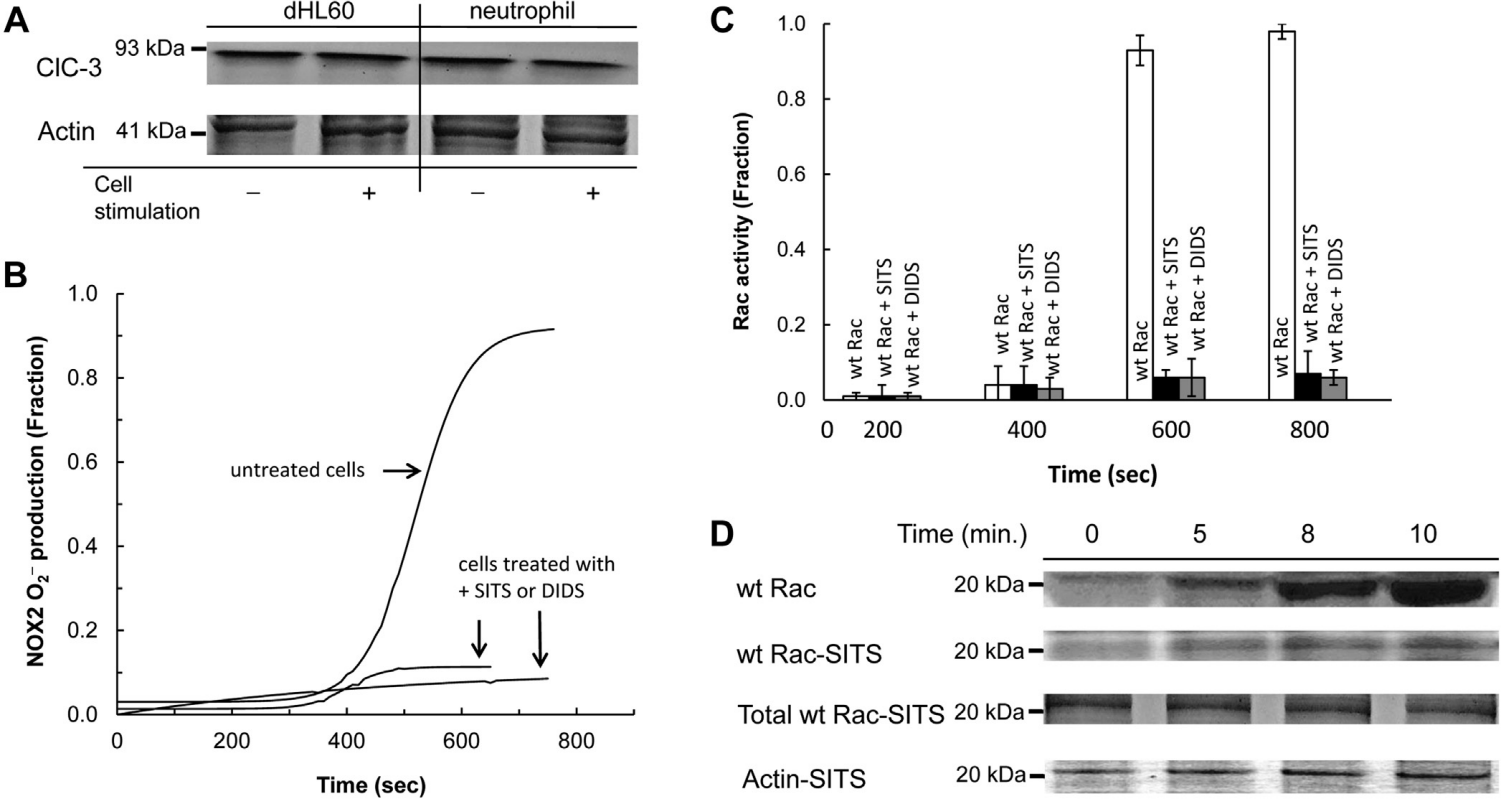
**Identification of ClC-3 on the plasma membrane and its relevance on the wt Rac assembly to the NOX2 complex in dHL60 cells.** The presence of ClC-3 on the plasma membrane and its function in the formation of the NOX2 complex was examined by using dHL60 cells. *A*, Western analyses were performed for the plasma membrane fractions of cells suspended in the sample buffer (see the Experimental procedures section) with the ClC-3 antibody (Alomone Labs). For this analysis, dHL60 cells unstimulated and stimulated with PMA for 10 min were used. As controls, the plasma membrane fractions of neutrophils unstimulated and stimulated with PMA for 10 min also were used. For loading controls, actin content in the whole-cell lysates was also examined. *B*, O_2_^−^ production from the PMA-stimulated dHL60 cells treated with and without SITS (100 μM) was examined by monitoring the oxidation of cytochrome *c* over time as described in the Experimental procedures section. As a control, another ClC-3 inhibitor, DIDS (100 μM), was used instead of SITS. *C*, Wt Rac activity in the PMA-stimulated, SITS-treated, and untreated dHL60 cells was examined as described in *C* of Figure 3, except that dHL60 cells were treated with and without the ClC-3 inhibitor SITS for 10 min before stimulation with PMA. *D*, the wt Rac binding to the NOX2 complex formation over time in the PMA-stimulated dHL60 cells in the presence and absence of ClC-3 inhibitor SITS (100 μΜ) was examined by using pull-down and Western analyses as described in *C*, the *middle panel* of Figure 3. Actin and total wt Rac content in the whole-cell lysates treated with the inhibitor were shown as loading controls. ClC-3, chloride channel-3; DIDS, 4,4-diisothiocyanatostilbene-2,2′-disulfonic acid; NOX2, NADPH oxidase 2; PMA, phorbol myristate acetate; SITS, 4-acetamide-4′-isothiocyanatostilbene-2,2′-disulfonic acid.

To explore this possibility, we examined the expression of ClC-3 and its potential function in the O_2_^−^-mediated activation of wt Rac activation and its effect on NOX2 function in cells. Figure 4*A* shows that the ClC-3 channel was ubiquitously expressed in the plasma membrane of dHL60 cells and human neutrophils. Their expression densities on the plasma membrane were unchanged even after the cells were stimulated (Fig. 4*A*). Minimal production of O_2_^−^ was observed when dHL60 cells were treated with either of the ClC-3 inhibitors, 4-acetamide-4′-isothiocyanatostilbene-2,2′-disulfonic acid (SITS) or 4,4-diisothiocyanatostilbene-2,2′-disulfonic acid (DIDS) (Fig. 4*B*). The effect of wt Rac activation on the dHL60 cells treated with SITS or DIDS was also minimal (Fig. 4*C*). Furthermore, wt Rac recruitment to the NOX2 phox complex to produce the active NOX2 complex was significantly impeded by the treatment of dHL60 cells with SITS (Fig. 4*D*) or DIDS (not shown). These results are intriguing because this appears to be the first report of the action of the ClC-3 inhibitors on wt Rac and NOX2 activation. Nevertheless, in taking into account that O_2_^−^ elicits a wt Rac redox response, these results suggest that ClC-3 functions as a O_2_^−^ conduit that enables cellular wt Rac activation by the extracellular O_2_^−^ and thus activation of NOX2 in cells. These results also suggest that ClC-3 is a key component for NOX2 activation in cells, a role not previously taken into account.

In considering the short life span of O_2_^−^, the question as to where and when there is enough O_2_^−^ to cross the plasma membrane and activate wt Rac arises. To address this question, we have analyzed the half-life and diffusion distance of O_2_^−^ under various cellularly relevant conditions (see details in the Supporting information section). Our analysis suggests that O_2_^−^ has a sufficiently long half-life to enable its diffusion to nearby cells, where it may be imported into the cell cytoplasm. The half-life of O_2_^−^ depends strongly on the concentration of its reaction partners, therefore its ability to act in this manner varies with cellular context. However, even at nanomolar O_2_^−^ concentrations, and in the presence of equimolar SOD, it is estimated that O_2_^−^ has a half-life of many tenths of a second enabling it to diffuse distances that span many cell diameters. Consequently, even at the beginning of NOX2 autoactivation (when O_2_^−^ concentration is low), enough O_2_^−^ is present to cross the plasma membrane through ClC-3 to hysteretically activate wt Rac. The H_2_O_2_ that is dismutated from O_2_^−^ can pass through aquaporins to reach the cellular NOX2 system (56, 64). However, because H_2_O_2_ has no effect on the hysteresis of the wt Rac and NOX2 activation over time (see the aforementioned section), the cellular influx of H_2_O_2_ is unlikely to be a factor in the cellular hysteresis of the wt Rac and NOX2 function over time.

### Rac-dependent autoactivation of NOX2 and its kinetic features

When summarized, the results of these cell-free and whole cell–based studies show that O_2_^−^ mediates the time-dependent hysteretic feedback loop activation between wt Rac and NOX2. Mechanically, although activation of one wt Rac activates only one NOX2, activation of one NOX2 generates many O_2_^−^ molecules, which activate many wt Rac proteins. Thus, the catalytic action of the activated NOX2 amplifies the cycle of the feedback loop activation events over time. Hysteresis as a function of time is a signature of the amplification of the feedback loop cycle over time, which is known to be autoactivation in enzyme function (53, 54, 55). Accordingly, the time-dependent hysteresis of the wt Rac and NOX2 activation is interpreted as a feature of the process of autoactivation of wt Rac and NOX2. Recognition of NOX2 autoactivation leads us to examine the NOX2 autoactivation–specific kinetic parameters, including the autoactivation rate constant (*k*_auto_) and reaction trigger threshold of active wt Rac (mole %) (see the Supporting information section); these describe the previously unknown kinetic features of NOX2 activation.

#### NOX2 autoactivation rate constant

**Figure 5:**
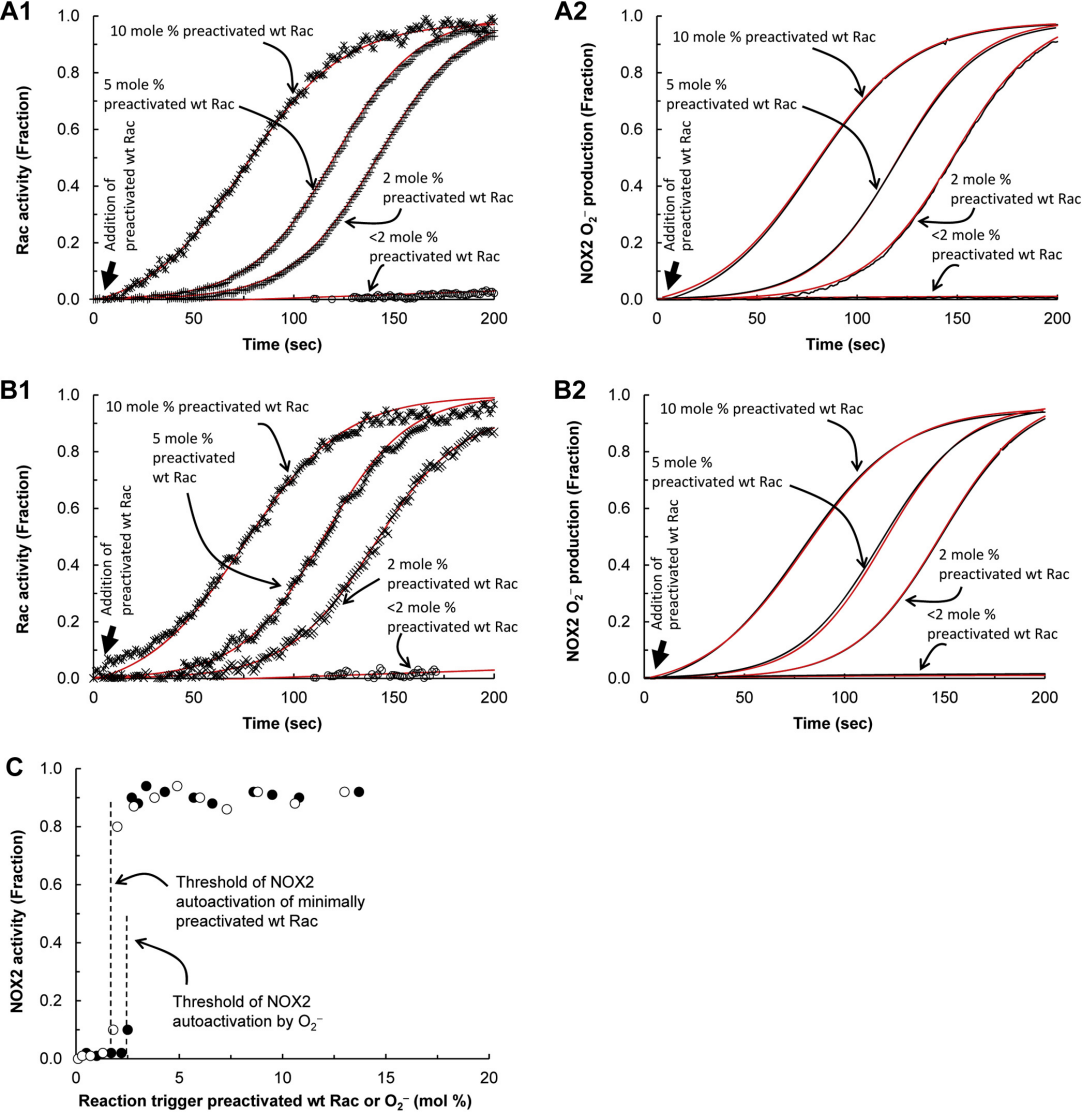
**Threshold concentrations of wt Rac and O**_**2**_^**−**^**for the initiation of NOX2 autoactivation.** NOX2 O_2_^−^ production in a cell-free system that contains the NOX2 phox complex and inactive wt Rac with various trigger amounts of active wt Rac or O_2_^−^ was measured by monitoring oxidation of cytochrome *c*. Wt Rac activation that corresponds to NOX2 O_2_^−^ production also was measured by using mant-GTP as described in the Experimental procedures section. The O_2_^−^ production and wt Rac activity-change profiles over time were fit to the autoactivation equation (see the Supporting information section, *red lines*), which gave the *k*_auto_ values for the NOX2 O_2_^−^ production as well as wt Rac activation that was triggered by the various fractions of active wt Rac or O_2_^−^. *A*1, the *k*_auto_ values for NOX2 O_2_^−^ production with <2, 2, 5, and 10 mol % active wt Rac trigger in the cell-free NOX2 system are, respectively, >15,000, 781, 509, and 383 s. *A*2, the corresponding *k*_auto_ values of the wt Rac activation, are, respectively, >15,000, 790, 511, and 389 s. *B*1, the *k*_auto_ values for the NOX2 O_2_^−^ production with <2, 2, 5, and 10 mol % of the trigger O_2_^−^ are, respectively, >15,000, 740, 445, and 369 s. *B*2, the corresponding *k*_auto_ values of the wt Rac activation, are, respectively, >15,000, 753, 467, and 344 s. *C*, the threshold concentrations of the active wt Rac and O_2_^−^ to trigger NOX2 autoactivation were determined by titration of the NOX2 phox complex containing inactive wt Rac in the cell-free system with titrants. The titrants were various concentrations of the active wt Rac (◯) and O_2_^−^ (●). The threshold concentrations of the active wt Rac and O_2_^−^ triggers for NOX2 autoactivation were determined, respectively, to be ∼2.0 and 2.5 mol % of active wt Rac with inactive wt Rac. NOX2, NADPH oxidase 2.

The parameter *k*_auto_ reflects the speed of NOX2 autoactivation. The *k*_auto_ values for NOX2 and wt Rac autoactivation in cell-free conditions were determined by using the autoactivation equation (see details in the Supporting information section). Each of the *k*_auto_ values of NOX2 autoactivation were nearly identical to the corresponding *k*_auto_ values of wt Rac autoactivation in the cell-free system (Fig. 5, *A*1, *A*2, *B*1 and *B*2). These results suggest that NOX2 autoactivation (or O_2_^−^ production) tightly couples with wt Rac autoactivation or *vice versa*.

**Figure 6:**
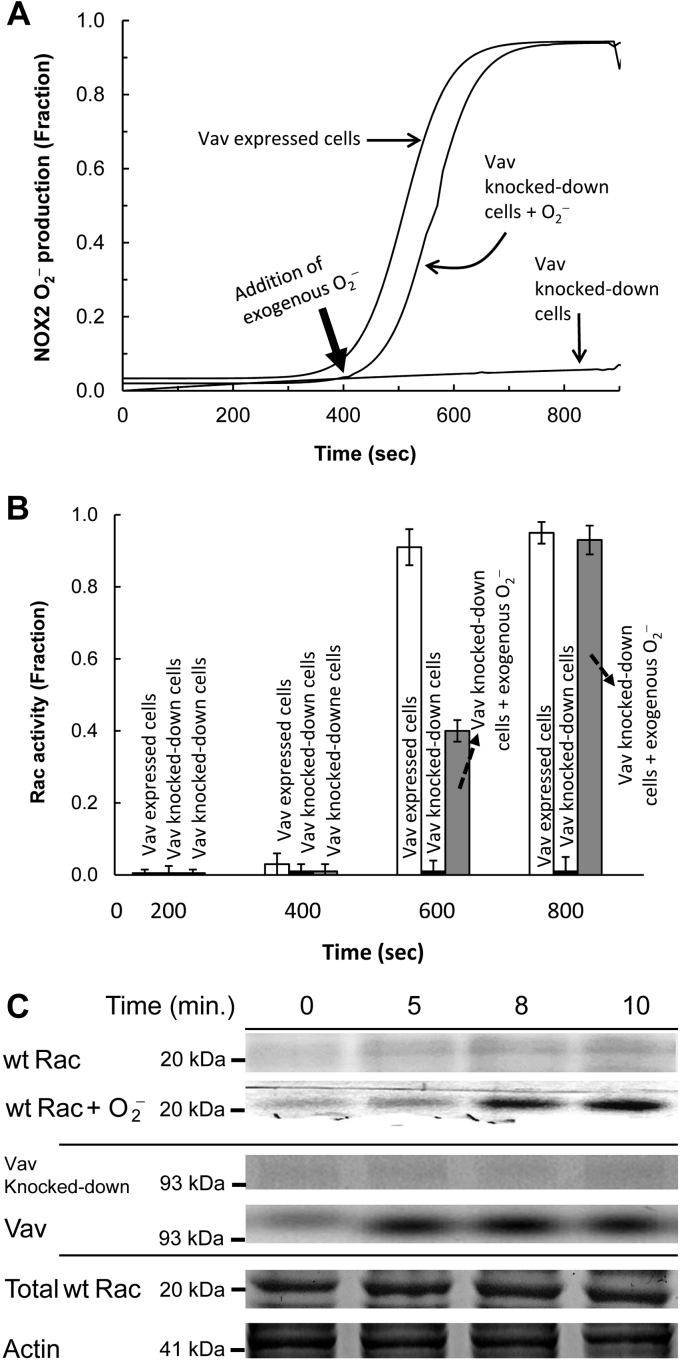
**Effect of Vav and O**_**2**_^**−**^**on the initiation of the NOX2 autoactivation.** The effect of Vav and exogenous O_2_^−^ on the initiation of activation of the hysteretic wt Rac and NOX2 was examined by using the Vav knocked-down dHL60 cells. Preparation and quantification of exogenous O_2_^−^ were described in the Experimental procedures section. *A*, O_2_^−^ production from the PMA-stimulated Vav knocked-down dHL60 cells in the presence and absence of exogenous O_2_^−^ (∼2 μM) was examined by monitoring the oxidation of cytochrome *c* over time as described in the Experimental procedures section. As a control, dHL60 cells that express Vav also were used. *B*, the wt Rac activity change in the PMA-stimulated Vav knocked-down dHL60 cells in the presence and absence of exogenous O_2_^−^ (∼2 μM) was as described in *B* of Figure 3. As a control, dHL60 cells that express Vav also were used. Three independent assays were performed, and the average values with SD are shown. *C*, *upper panel*, the wt Rac binding to the NOX2 phox complex in Vav knocked-down dHL60 cells over time in the presence and absence of exogenous O_2_^−^ was examined by using pull-down and Western analyses. *Middle panel*, lack of the Vav expression in the Vav knocked-down dHL60 cells also was examined by using Western analysis. For this analysis, the whole-cell lysate of Vav knocked-down dHL60 cells was used. As a control, a Vav Western analysis for dHL60 cells that express Vav also was used. *Lower panel*, total wt Rac and actin content in the whole-cell lysates of Vav knocked-down dHL60 cells are shown as loading controls. NOX2, NADPH oxidase 2; PMA, phorbol myristate acetate.

The *k*_auto_ value of NOX2 autoactivation in dHL60 cells (Fig. 6*A*) was also determined by using the autoactivation equation (not shown). The *k*_auto_ value of wt Rac autoactivation is unlikely to deviate from that of NOX2 autoactivation in dHL60 cells because the time-dependent activation kinetic profile of wt Rac (Fig. 6*B*) is exactly overlaid with that of NOX2 autoactivation in dHL60 cells (Fig. 6*A*). This analysis thus suggests that the tight coupling between NOX2 and wt Rac autoactivation is conserved in the cell system.

#### NOX2 autoactivation initiation threshold

A reaction trigger—a reaction primer or initiator—is necessary for initiation of NOX2 autoactivation (see details in the Supporting information section). This explains the requirement for a small fraction of the active wt Rac to trigger wt Rac activation and NOX2 O_2_^−^ production (see the NOX2 activation coupled with Rac activation section).

The minimal fraction of the active wt Rac that initiates NOX2 autoactivation is defined as the threshold for this event. In principle, O_2_^−^, as with active wt Rac, acting alone can initiate NOX2 autoactivation. This is because O_2_^−^ is capable of eliciting a wt Rac redox response in the absence of any Rho GEFs (38). To determine the NOX2 autoactivation threshold, its initiation was monitored after titration of the cell-free system with various concentrations of active wt Rac or O_2_^−^. The cell-free system used within this titration contained the NOX2 phox complex and inactive wt Rac but lacked the small fraction of preactivated wt Rac.

Figure 5*A*1 shows that, in the cell-free system, at least 2 mol % of the preactivated wt Rac trigger was required to initiate the hysteretic NOX2 O_2_^−^ production within 1 min. This result is consistent with the threshold value (2 mol % of active wt Rac) for the wt Rac trigger for initiation of NOX2 autoactivation determined in a cell-free system (Fig. 5*C*). Moreover, NOX2 O_2_^−^ production was essentially tied with hysteretic wt Rac activation at various concentrations of the additions of the preactivated wt Rac to the reaction mixture (Fig. 5*A*2).

Figure 5*B*1 shows that O_2_^−^ also was capable of initiating NOX2 autoactivation in 1 min in a cell-free system containing the NOX2 phox complex in the presence of inactive wt Rac and that at least 2 mol % of O_2_^−^ (mole % of O_2_^−^ with inactive wt Rac) was required to initiate NOX2 autoactivation. The kinetic profile of wt Rac autoactivation also links with that of the O_2_^−^-mediated NOX2 autoactivation because the *k*_auto_ values of wt Rac autoactivation are nearly identical to those of their corresponding NOX2 autoactivations (Fig. 5, *B*1 and *B*2). These results suggest that, as with wt Rac, O_2_^−^ can trigger NOX2 autoactivation that couples with the wt Rac autoactivation. Moreover, the amount of O_2_^−^ necessary to trigger NOX2 autoactivation in a cell-free system is similar to the amount required for triggering by wt Rac (Fig. 5*C*). This is hardly surprising because 1 mol of O_2_^−^ could generate stoichiometrically 1 mol of active wt Rac. Thus, it can be suggested that, regardless of how the trigger mechanism is produced, it initiates NOX2 autoactivation whenever its presence exceeds the required threshold.

As was the case in the cell-free system, a trigger may be necessary to initiate NOX2 autoactivation in the whole-cell system. Studies show that Vav is one of the Rho GEFs that functions to activate wt Rac in HL60 cells (65, 66). Accordingly, we examined whether Vav functions to produce active wt Rac that triggers NOX2 autoactivation in dHL60 cells.

Figure 6*A* shows that, unlike in the case of Vav-expressing dHL60 cells, minimal O_2_^−^ production occurred in Vav knocked-down dHL60 cells. Also, similar minimalism occurred with wt Rac activation (Fig. 6*B*) and its recruitment to the NOX2 phox complex (Fig. 6*C*) in Vav knocked-down dHL60 cells. These results suggest that in dHL60 cells, Vav functions to generate active wt Rac that, in turn, serves as the autoactivation reaction trigger. It also is notable that the Vav expression in dHL60 cells was completed at the early stage of NOX2 autoactivation, and then its expression level was unchanged over time (Fig. 6*C*). Moreover, the catalysis of enzymes, including Vav, follows a hyperbolic—but not hysteretic—function over time. When these occurrences are combined, we expect to observe a hyperbolic wt Rac activation over time in cells. However, this expectation is nevertheless incompatible with the observed hysteretic kinetic profile of wt Rac activation over time in cells (Fig. 6*B*). The best explanation for this incompatibility is that in dHL60 cells after Vav initiates NOX2 autoactivation, the redox-mediated wt Rac activation, instead of the Vav action, takes charge of NOX2 autoactivation and thus ends the contribution of Vav expression to overall NOX2 autoactivation.

In contrast to the case with the cell-free system, cells possess several other Rho GEFs as well as endogenous O_2_^−^-producing enzymes and systems (*e.g.*, mitochondrial respiration). Conversely, cells can consume O_2_^−^ by various quenching agents such as glutathione (GSH) and/or by many other redox-sensitive proteins such as Ras. Although the cell-free system lacks Rho GAPs that inactivate wt Rac, cells possess several Rho GAPs. Therefore, a quantitative evaluation of the threshold value determined in the cell-free system is challenged in the whole-cell system under our experimental conditions. However, an examination of the potential NOX2 autoactivation by a titration of the Vav knocked-down dHL60 cells with various amounts of exogenous O_2_^−^ still can ascertain whether exogenous O_2_^−^ is capable of initiating NOX2 autoactivation in the Vav knocked-down dHL60 cells. If it has this capability, titration can also indicate an apparent cellular threshold of the active wt Rac for the initiation of NOX2 autoactivation. It also should be noted that a determination of the exact concentration of the localized wt Rac that functions with the NOX2 system in cells is impossible under our experimental conditions. Thus, the value of the apparent threshold of the exogenous O_2_^−^ concentration that triggers NOX2 autoactivation in Vav knocked-down dHL60 cells can be expressed as molar (*e.g.*, micromolar) but not mole %.

Figure 6*A* also shows that O_2_^−^ alone successfully triggered NOX2 autoactivation in the Vav knocked-down dHL60 cells. This NOX2 autoactivation was tied with wt Rac activation (Fig. 6*B*) and its recruitment to the NOX2 phox complex (Fig. 6*C*) over time. Because Vav knocked-down dHL60 cells lacked the Vav needed to produce wt Rac, the active trigger, it can be interpreted that extracellular O_2_^−^ is capable of initiating cellular NOX2 autoactivation. The apparent threshold of O_2_^−^ for the initiation of NOX2 autoactivation was determined to be ∼2 μM in the Vav knocked-down dHL60 cells (detailed titrations are not shown). However, this apparent threshold cannot be interpreted as meaning that ∼2 μM is the sole threshold of O_2_^−^ for the initiation of cellular NOX2 autoactivation. This is because, as noted previously, other enzymes and cell systems can also produce active wt Rac that can possibly contribute to the exogenous O_2_^−^-mediated initiation of NOX2 autoactivation in Vav knocked-down dHL60 cells.

## Discussion

This study shows that the hysteretic NOX2 oxidative burst, an occurrence recognized for decades, is a reflection of NOX2 autoactivation. In explaining the mechanism of the time-dependent hysteresis of this oxidative burst, this study also explains the physiology and pathophysiology of NOX2 associated with the NOX2 oxidative burst.

### Mechanistic features of NOX2 autoactivation

On the basis of the results shown in this study, we propose a mechanism for the action of the positive feedback loop between Rac and NOX2 that renders the hysteretic NOX2 oxidative burst as follows: (i) the time-dependent feedback loop of the Rac redox response: Rac is feedback activated by the O_2_^−^ produced from NOX2, (ii) the active Rac-binding interaction with the NOX2 phox complex: the activated Rac binds to the NOX2 phox complex to produce active NOX2; and (iii) amplification of the feedback loop of the Rac and NOX2 activation cycle: the positive feedback cycle of activation between Rac and NOX2 is amplified over time until NOX2 is fully activated. Details of each step are discussed later.

#### Time-dependent feedback loop of Rac redox response

Mechanistically, the Rac redox response enables the O_2_^−^ produced from NOX2 to activate more of Rac. This O_2_^−^-dependent Rac redox feature was reported previously (38), but how it contributed to NOX2 functions was unknown.

The previous study (38) shows that O_2_^−^ facilitates the dissociation of the bound nucleotide to produce a nucleotide-deficient Rac (apo-Rac). The study also shows that when there was an excess O_2_^−^ (50–100 μM), a fresh nucleotide was still capable of binding back to apo-Rac to produce a nucleotide-bound Rac. A radical quencher, such as ascorbic acid, greatly enhanced this binding process. Our recent study shows that without an excess of O_2_^−^ (*e.g.*, <50 μM), the binding of a fresh nucleotide to apo-Rac to produce a nucleotide-bound Rac was up to fivefold faster than with an excess of O_2_^−^ concentration (not shown). However, the effect of the radical quencher on the fresh nucleotide association with apo-Rac was nevertheless conserved, even without an excess of O_2_^−^ (*e.g.*, <50 μM, not shown). The whole-cell system contains many radical quenchers such as GSH. Thus, cellular radical quenchers may be involved in the Rac redox response in dHL60 cells.

Within this study, we showed that the presence of the radial quencher in the cell-free NOX2 assay system further enhanced the hysteretic Rac activation and thus NOX2 O_2_^−^ production over time. This is consistent with the previous study showing that the radical quencher enhanced the binding of the fresh nucleotide to apo-Rac to produce a nucleotide-bound Rac (38). However, the effect of the radical quencher on the hysteretic Rac and NOX2 activation over time is noticeable but not significant. We interpreted these results as that the initial and maximal O_2_^−^ production from NOX2 during the assay period was not significantly high as in the previous study (38). Therefore, even without the presence of the radical quencher, the apo-Rac produced by the action of O_2_^−^ is capable to bind to GTP to produce an active GTP-bound Rac. Our experimental condition such as the use of an excess of fresh GTP (*i.e.*, 10 mM) could also facilitate the production of active GTP-bound Rac without the radical quencher. Consequently, the production of active GTP-bound Rac without the radical quencher leaves a little room for the radical quencher to further enhance the binding of the apo-Rac to GTP to produce an active GTP-bound Rac. These analyses overall explain the minimal effect of the radical quencher on the production of active GTP-bound Rac in the cell-free assay system. The production of active GTP-bound Rac without the radical quencher also explains the previous study in which a NOX2 oxidative burst was observed in a cell-free system without the presence of a radical quencher (44).

#### Active Rac-binding interaction with the NOX2 phox complex

Studies have shown that the Rac effector and/or insert region–binding interactions with the NOX2 phox complex are critical for NOX2 activation (22, 25, 26, 28, 29, 30). This study shows that these Rac NOX2-binding interactions do not cause the hysteretic Rac and NOX2 activation over time; instead, they merely allow the binding of the hysteretically activated Rac to the NOX2 phox complex. Hysteretic NOX2 activation over time has been attributed to the time dependent-amplification feature of the NOX2 autoactivation (see later).

#### Amplification of the feedback loop of Rac and NOX2 activation cycle

The time-dependent hysteresis of the NOX2 oxidative burst cannot be explained solely by the notion of a positive feedback loop acting between Rac and NOX2. This is because the action of the positive feedback loop does not necessarily occur through the hysteretic mode over time. The action of the positive feedback loop can be amplified, lessened, or even steadied over time. However, only amplification over time yields the time-dependent hysteresis. Thus, the hysteretic NOX2 oxidative burst is interpreted in terms of the time-dependent amplification of the action of the positive feedback loop between Rac and NOX2. This amplification results in increased production of the active NOX2 over time.

The amplification of the action of the positive feedback loop as a function of time enables automation of enzyme activation (self-driven enzyme activation), which is termed autoactivation. It must be emphasized that, without the amplification feature, the action of a positive feedback loop alone does not yield autoactivation of the enzyme function over time. This is because the time-dependent amplification of the action of the positive feedback loop is the key driving force of automation of the enzyme activation. Accordingly, the NOX2 oxidative burst, a reflection of Rac and NOX2 autoactivation, is a result of the combination of the action of the positive feedback loop and its amplification over time.

### Time-dependent allostery of the NOX2 activity regulation

From the viewpoint of regulation of enzymatic activity, Rac is an allosteric ligand of NOX2. The finding of the NOX2 autoactivation feature suggests that the allosteric regulation of NOX2 function with Rac occurs through the time-dependent amplification of the feedback loop on Rac activation. However, this time-dependent feature of the NOX2 allostery differs from conventional enzyme allostery. This difference is because it functions with the ligand concentration that yields a sigmoidal curve over the ligand concentration (67, 68, 69, 70).

The time-dependent allostery found in NOX2 is also found in other enzymes such as SOS (53, 55). In the case of SOS, the SOS allosteric ligand Ras is also the substrate for SOS catalysis; thus, the time-dependent SOS allostery is classified as homotropic (53, 55). However, the NOX2 allosteric ligand wt Rac is not the substrate for NOX2 catalysis; thus, the time-dependent NOX2 allostery is classified as heterotropic.

### Advantage of the NOX2 autoactivation and its implication

There are two autoactivation-specific kinetic parameters: (i) the autoactivation rate constant and (ii) the trigger threshold concentration of the autoactivation cycle. Evaluation of these autoactivation-specific kinetic parameters associated with the NOX2 function provides insight into the previously unknown features of regulation of NOX2 activity.

The value of the autoactivation constant, *k*_auto_, represents how fast autoactivation can be completed over time to reach maximal enzyme activity. This study shows that the *k*_auto_ value of NOX2 is similar to that of Rac. The active Rac-binding interaction with the NOX2 phox complex to produce active NOX2 is the key step in linking Rac autoactivation to NOX2 autoactivation. Therefore, the *k*_auto_ value similarity can be interpreted to mean that the binding process of the active Rac to the NOX2 phox complex to produce active NOX2 does not deter or alter the rate of Rac autoactivation delivered to the rate of the NOX2 autoactivation. This notion is supported by the lack of an effect by either the Rac-effector or Rac-insert region mutants on NOX2 autoactivation.

The *k*_auto_ values of Rac and NOX2 estimated within this study suggest that the rate of Rac and NOX2 autoactivation is sufficiently fast. In cells, Rac and thus NOX2 autoactivation enable NOX2 to populate its fully active form in 1 min. Our *in vitro* kinetic analysis estimates that Vav, one of the Rho GEFs, must be overexpressed up to 1000-fold compared with the normal Vav expression in cells to achieve the activation of Rac and thus NOX2 in 1 min (not shown). Thus, this analysis suggests that the Rac and NOX2 autoactivation are sufficiently efficient to produce fully active NOX2 without overexpression of the RhoGEF Vav. Overexpression of a protein involves the operation of various cellular machinery, including ribosomes. This justifies the speculation that cells can avoid such resource-intensive operations by using autoactivation kinetics.

In contrast to the conventional enzyme activation, the process of enzyme autoactivation requires a trigger. Within NOX2 autoactivation, active Rac has been identified as the trigger. We determined that a 2 mol % concentration of active Rac is required as a threshold amount to trigger NOX2 activation in a cell-free system. The overall contribution of the trigger and autoactivation of NOX2 on the fraction of the activated NOX2 in a cell-free system is estimated to be as follows: of the total activated NOX2 (100%), the 2 mol % trigger for active wt Rac is responsible for 2% of activated NOX2, and the remaining 98% of the activated NOX2 was self-activated during NOX2 autoactivation. In other words, NOX2 autoactivation is responsible for the production of 98% of active NOX2, whereas enzymatic NOX2 activation only accounts for 2% of the active NOX2. When combined with the fast NOX2 autoactivation rate (see previous one), this analysis further suggests that NOX2 autoactivation allows for the rapid buildup of a large quantity of active NOX2 compared with that of the enzymatic NOX2 activation process. We speculate that these features of NOX2 autoactivation are advantageous for combating infections where the fast and robust response of NOX2 is needed.

Unlike the situation with the cell-free system, our experimental conditions did not allow us to design a whole-cell system entirely free of the NOX2 autoactivation trigger. This NOX2 autoactivation trigger–free condition is a prerequisite to performing a titration of NOX2 with a trigger to determine the NOX2 trigger threshold in cells. For example, even in the case in which dHL60 cells lack Vav, the cells still could express other types of Rho GEFs and/or O_2_^−^-producing enzymes as well as have systems capable of producing the active Rac trigger. Therefore, we were unable to measure the threshold for a cellular trigger for NOX2 autoactivation. Accordingly, verification or comparison of a threshold for the trigger of a cell-free system with the cellular trigger threshold, the NOX2 autoactivation was not feasible. Nevertheless, our cell studies show no initiation of NOX2 autoactivation occurred when Vav was knocked down in dHL60 cells. This result suggests the necessity of the NOX2 autoactivation trigger in the whole-cell system and that Vav serves as the trigger for active Rac production. This study also shows that, in the dHL60 cells, Vav expression was completed in the early stage of the NOX2 autoactivation process but did not produce enough activated NOX2 as fast as needed. Thus, as in the case of the cell-free system, only a minimal amount of active Rac—produced by Vav—is required for overall NOX2 activation, and the largest fraction of activated NOX2 is self-activated by the NOX2 autoactivation process and not by the Vav catalytic action in the dHL60 cells. However, because of the several different forms of Rho GEFs expressed in different cells, it remains possible that other Rho GEFs in other cells may also produce triggers. Given that the producer of a trigger, such as Rho GEFs, is critical to initiate NOX2 autoactivation, it could be tightly regulated in the whole-cell system. The implication of tightly regulating the producer of the trigger for NOX2 autoactivation is likely one of the previously underevaluated significances of the regulation of NOX2 activity in cells.

As we have also shown, an extracellular redox agent such as O_2_^−^ was also capable of initiating NOX2 autoactivation. The apparent threshold value of O_2_^−^ to initiate NOX2 autoactivation in the Vav knocked-down dHL60 cells also was determined to be ∼2 μM. However, this value does not necessarily indicate that ∼2 μM O_2_^−^ is capable of initiating NOX2 autoactivation in a whole-cell system. This is because the experimental conditions that gave this apparent value did not entirely exclude other potential O_2_^−^ and active wt Rac producers in the Vav knocked-down dHL60 cells. Nevertheless, this finding opens the possibility for NOX2 autoactivation to occur at the level of cell clusters. For example, as an immune response, the initiation of NOX2 autoactivation occurs only in a few selected neutrophils in the neutrophil clusters. The O_2_^−^ produced from these activated neutrophils then initiates NOX2 autoactivation in other neutrophils of the neutrophil clusters. Further studies also are necessary to clarify the potential immune defense system associated with NOX2 autoactivation at the level of cell clusters.

### Role of chloride channel in the NOX2 activation

The plasma membrane spatially separates the cellular NOX2 subunits, including Rac, from the extracellularly released O_2_^−^. Therefore, the extracellular O_2_^−^ released from NOX2 cannot directly target and activate cellular Rac. Previous studies showed that the chloride channel, ClC-3, functions as a O_2_^−^ conduit (61, 62, 63). Our study shows that ClC-3 in fact enables the extracellularly released O_2_^−^ to pass through the plasma membrane to target and activate Rac.

Note that there were no complete blockages of the NOX2 autoactivation in dHL60 cells, despite their treatment with an excess of ClC-3 inhibitor, SITS or DIDS. We speculate that an unknown O_2_^−^ channel(s) or transport system(s) may exist, which is unaffected by the ClC-3 inhibitors. It also is possible that other potential cellular O_2_^−^ producers or systems (*e.g.*, mitochondrial respiration) could contribute to NOX2 autoactivation even as SITS or DIDS blocks the influx of O_2_^−^ through ClC-3. Further studies are necessary to identify the mechanism by which these C1C-3 inhibitors achieve this incomplete blockage of cellular NOX2 autoactivation.

### Autoactivation of NOX1

This hysteretic NOX2 oxidative burst is also observed with NOX1 but not with other NOX family proteins such as NOX4 (not shown). The common denominator between NOX2 and NOX1 seems to be that they both require Rac for their activity. However, NOX1 and NOX2 differ in their subunits and structural features. In fact, the active Rac of NOX2 apparently has a lower initiation threshold than NOX1 (not shown). We suspect that the difference may lie in unique subunit features specific to NOX2 and NOX1. Further studies that clarify the mechanistic and potential functional differences between NOX2 and NOX1 autoactivation are necessary.

### Potential diseases associated with the deregulation of NOX2 and NOX1 autoactivation

This study shows that the role of active Rac as a reaction trigger is a key factor in the regulation of NOX2 autoactivation. Therefore, any potential deregulation of the expression of, or function of, the producers of NOX2 autoactivation triggers, such as Vav or a foreign-origin redox agent, could lead to uncontrolled population of activated NOX2 in cells. NOX1 is expressed in a broader range of cell types and organs than NOX2; therefore, the potential deregulation of NOX1 autoactivation may link to a broad range of diseases. Accordingly, further studies are necessary to examine these possibilities. Given that ClC-3 plays a key role in the influx of O_2_^−^ into cells, the use of ClC-3 inhibitors may be a potential therapeutic for the deregulation of the foreign-origin O_2_^−^-mediated initiation of NOX1 and NOX2 autoactivation.

## Experimental procedures

Cell-free and cell-based NOX2 functional analyses were performed to examine the hysteresis of NOX2 O_2_^−^ production over time and its significance in the regulation of NOX2 function. All kinetic measurements were fractionized by dividing them by the maximal value within the assay.

### Preparation of chemicals and enzymes

Unless otherwise noted, the chemicals used for all experiments were of the highest grade. O_2_^−^ was prepared from KO_2_ (solid power > 96%; Sigma). KO_2_ was dissolved in a serum stoppered assay vial containing dimethyl sulfoxide, and the O_2_^−^ content in dimethyl sulfoxide was determined by using the luminol-based assay kit (Sigma). Note that, according to the vendor, the main impurities of KO_2_ are K_2_O_2_ and KOH. Both K_2_O_2_ and KOH in water generate a hydroxide ion (OH^−^) base, which is highly reactive and thus nullified by a nonspecific reaction with the buffer components used in the cell-free assay system. Thus, the results of the kinetic studies using O_2_^−^ generated from KO_2_ can be solely attributed to the effect of O_2_^−^. H_2_O_2_ (30% w/w; Sigma) was diluted with double-distilled water to 10% (w/w) prior to use. SOD, mitogen-activated protein (MAP) kinase, and protein kinase C (lyophilized; Sigma) were used after being dissolved in an assay buffer containing 10 mM MgCl_2_, 50 mM KCl, 1 mM NaCl, 0.2 mM NADPH, and 10 mM GTP in 10 mM Mops (pH 7.4).

### Preparation of the cell-free NOX2 phox subunits

Cytochrome b558 was purified from the human embryonic kidney 293 cell line that stably expresses NOX2 with p22 by heparin with hydroxyapatite chromatography, which was then relipidated and reflavinated as described in the previous study (71, 72). This highly purified unlabeled cytochrome b558 contained only gp91 and p22.

When necessary, rhodamine-tagged cytochrome b558 was used instead of the unlabeled cytochrome b558. Briefly, the rhodamine-tagged cytochrome b558 was produced as follows: to avoid nonspecific rhodamine tags on the NOX2 component–binding interfaces, rhodamine B (100 μM) was used to treat, as described previously (73), the fully assembled and actively O_2_^−^-producing NOX2 complexes in the cell-free system. The rhodamine-treated NOX2 sample was then dialyzed against an SDS-free Tris–HCl buffer containing a high salt concentration (500 mM NaCl, pH 7.4) to disassemble the rhodamine-tagged NOX2 components from cytochrome b558. The dialyzed sample was then filtered with an Amicon Centricon concentrator (100 kDa cut off) to collect only the rhodamine-tagged cytochrome b558. Finally, a size-exclusion FPLC using a Superose 6 column was performed to obtain rhodamine-tagged cytochrome b558 (>95% pure; see the Supporting information section).

All the soluble *phox* subunits for NOX2 were expressed in, and purified from, Sf9 insect cells (47). Before their use, p47 and p67 were phosphorylated with MAP kinase and protein kinase C as described previously (23). These phox subunits (>95% pure; see the Supporting information section) were separated from MAP kinase and protein kinase C by using a size-exclusion FPLC equipped with a Superose 6 column.

### Preparation of the active and inactive Rac

Wt Rac and its redox inert mutants (C18S and C157 Rac), effector region mutants (A27K and G30S Rac), and inert region mutants (K132E and L134R Rac) were expressed in, and purified from, *Escherichia coli*. Depending on the purification conditions, as-purified Rac typically consists of a <5% GTP-bound active form and a >95% GDP-bound inactive form. To produce a nearly complete inactive Rac fraction (>99.9% GDP-bound Rac), the as-purified Rac was incubated with an excess of GDP (>10 mM) in an exchange buffer containing 0.1 mM MgCl_2_ and 1 mM NaCl in 10 mM Mops (pH 7.4) for 10 h at room temperature and then was applied onto the size-exclusion FPLC column. The eluent containing the GDP-bound active Rac (>95% pure; see the Supporting information section) was further washed with the assay buffer by using an Amicon Centricon concentrator (30 kDa cut off). To prepare the preactivated Rac, the as-purified Rac was incubated with an excess GTP (>10 mM) and ammonium sulfate (400 mM) in the exchange buffer for 1 h and then was applied onto the size-exclusion FPLC column. The eluent containing the GTP-bound active Rac (>95% pure; see the Supporting information section) was further washed with the assay buffer by using an Amicon Centricon concentrator (30 kDa cut off).

### *In vitro* cell-free NOX2 functional analyses

A modified version of the cell-free NOX2 system (47, 74) was used to examine the *in vitro* kinetic features of NOX2 O_2_^−^ production over time. The modifications were (i) the use of a heparin–hydroxyapatite–based highly purified cytochrome b558 (gp91 plus p22) instead of the cell-membrane extract and (ii) the addition, when necessary, of a fraction of the preactivated wt Rac (see the later section) to the cell-free assay system. The modified NOX2 assay system thus consists of the highly purified cytochrome b558 (gp91 and p22) and the cytosolic phox subunits of NOX2 (p40, p47, and p67) in an assay buffer. The addition of SDS (100 μM) to the cell-free system produces the NOX2 phox complex, comprised of cytochrome b558, p40, p47, and p67 (Fig. 1). The final concentrations of cytochrome b558 for all the other cytosolic NOX2 phox subunits were 100 nM.

The O_2_^−^ production from the cell-free NOX2 system was determined by monitoring the reduction of cytochrome *c* as essentially described in the previous study (47, 74). The cell-free system containing the NOX2 phox complex was incubated with cytochrome *c* (100 μM) for 2 min; wt or mutant Rac (1 μM) was then added to initiate the reaction. When necessary, a small fraction of preactivated wt Rac was added to the assay system before adding wt or mutant Rac. For example, >2 mol % of the preactivated wt Rac was a prerequisite to observing the NOX2 O_2_^−^ production with the inactive Rac sample (>99.9% GDP-bound Rac) within the assay period (*e.g.*, within 1 min). This was determined to be because NOX2 activation with inactive wt Rac requires preactivated wt Rac as a trigger (see the Supporting information section). The change in the time-dependent reduction of cytochrome *c* was monitored with a Thermomax microplate reader (Molecular Devices).

The cell-free NOX2 system was further modified to examine the *in vitro* NOX2 complex formation over time. Rhodamine-tagged cytochrome b558 was used to produce the rhodamine-tagged NOX2 phox complex in the cell-free system. Within this modified system, the binding of phox subunits and wt Rac to the rhodamine-tagged cytochrome b558 results in an increase in the intensity of the rhodamine fluorescence. This change in rhodamine fluorescence can be monitored simultaneously with NOX2 O_2_^−^ production over time. To initiate the reaction, as with the O_2_^−^ production assay, wt Rac was added to the cell-free NOX2 system containing the rhodamine-tagged NOX2 phox complex. The change in the time-dependent rhodamine fluorescence intensity was monitored with a fluorescence spectrometer (LS 55; PerkinElmer).

For the *in vitro* wt Rac kinetic assays, the fluorescent probe *N*-methyl-3′-O-anthranoyl (mant)–tagged nucleotide (*e.g.*, mant-GTP) was used (16, 53, 75, 76, 77, 78). To examine the change in wt Rac activity in the NOX2 O_2_^−^ production and complex formation, mant-GTP instead of unlabeled GTP was added to the cell-free NOX2 system. The binding of mant-GTP to wt Rac, producing active wt Rac, results in an increase in mant fluorescence, which was monitored over time with a fluorescence spectrometer (LS 55).

### Preparation of dHL60 cells

Human HL60 cells and their variant forms were grown in an RPMI1640 medium containing low glucose (5.5 mM) and 10% fetal bovine serum at 37 °C in a 5% CO_2_ atmosphere; the media were replaced every 2 days. HL60 cells were differentiated into neutrophil-like cells (dHL60) by adding all-*trans*-retinoic acid (1 μM) to the media for 5 days as described in the previous studies (79, 80). When necessary, NOX2 activation was initiated by treating dHL60 cells with phorbol myristate acetate (PMA; 1 μM).

The redox-inert C18S or C157S Rac- or constitutively active G12V Rac-expressing HL60 cells were generated as follows. Both wt Rac1 and Rac2 were first knocked down in HL60 cells by using lentivirus-mediated shRNAs. These shRNAs target the 3′ untranslated regions of the endogenous wt Rac1 and Rac2 in HL60 cells (Qiagen). The Rac knockdowns were validated by using quantitative real-time PCR. The HL60 cells were then stably transfected with the C18S or C157S Rac or G12V Rac construct in the pMSCV vector according to the specifications of the manufacturer (Thermo Fisher Scientific). Vav knocked-down HL60 cells were generated using lentivirus-mediated shRNAs that target the 3′ untranslated regions of the endogenous Vav1 and Vav2. The Vav knockdowns were validated by using quantitative real-time PCR. When necessary, human neutrophils also were used as a control; these were obtained from a single individual and Astarte Biologics (73).

### Whole-cell NOX2 functional analyses

The O_2_^−^ production from the cells also was determined by monitoring the cytochrome *c* reduction as essentially described in the previous study (47, 74). Briefly, cells (∼5 × 10^5^ cells) in a Krebs–Ringer glucose buffer were incubated with cytochrome *c* (100 μM) for 2 min, and the stimuli PMA was added to initiate NOX2 activation. The change in the time-dependent cytochrome *c* reduction was monitored with a Thermomax microplate reader as described for the cell-free assay. When necessary, cells were treated with exogenous O_2_^−^ followed by treatment with PMA. Exogenous O_2_^−^ was prepared from KO_2_ (Sigma). The content of O_2_^−^ in the assay solution was determined by using a luminol-based assay kit (Sigma).

The cellular active NOX2 complex formation was examined by using pull-down and Western analyses. The PMA-stimulated dHL60 cells (∼5 × 10^5^ cells) were chilled on ice at specific time points and then lysed using a sonicator in lysis buffer containing 1 mM DTT, 1 mM PMSF, 0.1 mM EDTA, 5 mM MgCl_2_, and 500 mM NaCl in 50 mM Tris–HCl (pH 7.4). The whole-cell lysates were ultracentrifuged to collect plasma membrane fractions. These fractions were suspended in a sample buffer containing 5 mM MgCl_2_ and 2% Triton X-100 in 50 mM Tris–HCl (pH 7.4). The NOX2 complex was then pulled down with magnetic agarose beads tagged with the monoclonal gp91 antibody (Anti-gp91-Phox Antibody; Sigma). The pull-down samples were resuspended with the sample buffer and then were used for the Western analyses with various NOX2 subunit antibodies (anti-p40, anti-p47, and anti-67-phox antibodies; Sigma) as well as the Rac1 monoclonal antibody (Thermo Fisher Scientific). These antibodies are highly efficacious and specific, according to vendors.

When necessary, the phosphorylation quantity of NOX phox subunits was quantified by spraying malachite green (0.0001% w/v) in a buffer containing 5 mM MgCl_2_ and 0.1 mM EDTA in 50 mM Tris–HCl (pH 7.4) on the phox subunit Western blot membrane and then heating it at 200 °C for 2 min. Densitometry was used to determine the relative phosphorylation band density. The status of wt Rac activity within the pull-down samples also was examined by using a colorimetric wt Rac activity assay kit (Abcam). This assay uses the p21-activated kinase 1 p21-binding domain, which specifically binds only to active wt Rac. All wt Rac activity assays were performed independently three times.

## Data availability

All data are contained within the article and supporting information.

## Supporting information

This article contains supporting information.

## Conflict of interest

The authors declare that they have no conflicts of interest related to the content of this article.

## References

[1] Brandes R.P., Kreuzer J. (2005). Vascular NADPH oxidases: Molecular mechanisms of activation. Cardiovasc. Res..

[2] Roy K., Wu Y., Meitzler J.L., Juhasz A., Liu H., Jiang G., Lu J., Antony S., Doroshow J.H. (2015). NADPH oxidases and cancer. Clin. Sci..

[3] Panday A., Sahoo M.K., Osorio D., Batra S. (2015). NADPH oxidases: An overview from structure to innate immunity-associated pathologies. Cell. Mol. Immunol..

[4] Streeter J., Thiel W., Brieger K., Miller F.J. (2013). Opportunity nox: The future of NADPH oxidases as therapeutic targets in cardiovascular disease. Cardiovasc. Ther..

[5] Cifuentes-Pagano E., Meijles D.N., Pagano P.J. (2014). The quest for selective nox inhibitors and therapeutics: Challenges, triumphs and pitfalls. Antioxid. Redox Signal..

[6] Wang H., Hartnett M.E. (2017). Roles of nicotinamide adenine dinucleotide phosphate (NADPH) oxidase in angiogenesis: Isoform-specific effects. Antioxidants (Basel).

[7] Thomas D.C. (2017). The phagocyte respiratory burst: Historical perspectives and recent advances. Immunol. Lett..

[8] Parkos C.A., Dinauer M.C., Jesaitis A.J., Orkin S.H., Curnutte J.T. (1989). Absence of both the 91kD and 22kD subunits of human neutrophil cytochrome b in two genetic forms of chronic granulomatous disease. Blood.

[9] Dinauer M.C., Pierce E.A., Bruns G.A., Curnutte J.T., Orkin S.H. (1990). Human neutrophil cytochrome b light chain (p22-phox). Gene structure, chromosomal location, and mutations in cytochrome-negative autosomal recessive chronic granulomatous disease. J. Clin. Invest..

[10] Levy R., Dana R., Leto T.L., Malech H.L. (1994). The requirement of p47 phosphorylation for activation of NADPH oxidase by opsonized zymosan in human neutrophils. Biochim. Biophys. Acta.

[11] Forbes L.V., Moss S.J., Segal A.W. (1999). Phosphorylation of p67phox in the neutrophil occurs in the cytosol and is independent of p47phox. FEBS Lett..

[12] Hoyal C.R., Gutierrez A., Young B.M., Catz S.D., Lin J.H., Tsichlis P.N., Babior B.M. (2003). Modulation of p47PHOX activity by site-specific phosphorylation: Akt-dependent activation of the NADPH oxidase. Proc. Natl. Acad. Sci. U. S. A..

[13] Dang P.M., Cross A.R., Quinn M.T., Babior B.M. (2002). Assembly of the neutrophil respiratory burst oxidase: A direct interaction between p67PHOX and cytochrome b558 II. Proc. Natl. Acad. Sci. U. S. A..

[14] Dang P.M., Morel F., Gougerot-Pocidalo M.A., El Benna J. (2003). Phosphorylation of the NADPH oxidase component p67(PHOX) by ERK2 and P38MAPK: Selectivity of phosphorylated sites and existence of an intramolecular regulatory domain in the tetratricopeptide-rich region. Biochemistry.

[15] Colicelli J. (2004). Human RAS superfamily proteins and related GTPases. Sci. STKE.

[16] Abe K., Rossman K.L., Liu B., Ritola K.D., Chiang D., Campbell S.L., Burridge K., Der C.J. (2000). Vav2 is an activator of Cdc42, Rac1, and RhoA. J. Biol. Chem..

[17] Wennerberg K., Rossman K.L., Der C.J. (2005). The Ras superfamily at a glance. J. Cell Sci..

[18] Matono R., Miyano K., Kiyohara T., Sumimoto H. (2014). Arachidonic acid induces direct interaction of the p67(phox)-Rac complex with the phagocyte oxidase Nox2, leading to superoxide production. J. Biol. Chem..

[19] Han C.H., Freeman J.L., Lee T., Motalebi S.A., Lambeth J.D. (1998). Regulation of the neutrophil respiratory burst oxidase. Identification of an activation domain in p67(phox). J. Biol. Chem..

[20] Koga H., Terasawa H., Nunoi H., Takeshige K., Inagaki F., Sumimoto H. (1999). Tetratricopeptide repeat (TPR) motifs of p67(phox) participate in interaction with the small GTPase Rac and activation of the phagocyte NADPH oxidase. J. Biol. Chem..

[21] Lapouge K., Smith S.J., Walker P.A., Gamblin S.J., Smerdon S.J., Rittinger K. (2000). Structure of the TPR domain of p67phox in complex with Rac.GTP. Mol. Cell.

[22] Diebold B.A., Bokoch G.M. (2001). Molecular basis for Rac2 regulation of phagocyte NADPH oxidase. Nat. Immunol..

[23] Dang P.M., Cross A.R., Babior B.M. (2001). Assembly of the neutrophil respiratory burst oxidase: A direct interaction between p67PHOX and cytochrome b558. Proc. Natl. Acad. Sci. U. S. A..

[24] Dang P.M., Johnson J.L., Babior B.M. (2000). Binding of nicotinamide adenine dinucleotide phosphate to the tetratricopeptide repeat domains at the N-terminus of p67PHOX, a subunit of the leukocyte nicotinamide adenine dinucleotide phosphate oxidase. Biochemistry.

[25] Freeman J.L., Kreck M.L., Uhlinger D.J., Lambeth J.D. (1994). Ras effector-homologue region on Rac regulates protein associations in the neutrophil respiratory burst oxidase complex. Biochemistry.

[26] Sarfstein R., Gorzalczany Y., Mizrahi A., Berdichevsky Y., Molshanski-Mor S., Weinbaum C., Hirshberg M., Dagher M.C., Pick E. (2004). Dual role of Rac in the assembly of NADPH oxidase, tethering to the membrane and activation of p67phox: A study based on mutagenesis of p67phox-Rac1 chimeras. J. Biol. Chem..

[27] Miyano K., Koga H., Minakami R., Sumimoto H. (2009). The insert region of the Rac GTPases is dispensable for activation of superoxide-producing NADPH oxidases. Biochem. J..

[28] Freeman J.L., Abo A., Lambeth J.D. (1996). Rac “insert region” is a novel effector region that is implicated in the activation of NADPH oxidase, but not PAK65. J. Biol. Chem..

[29] Nisimoto Y., Freeman J.L., Motalebi S.A., Hirshberg M., Lambeth J.D. (1997). Rac binding to p67(phox). Structural basis for interactions of the Rac1 effector region and insert region with components of the respiratory burst oxidase. J. Biol. Chem..

[30] Price M.O., Atkinson S.J., Knaus U.G., Dinauer M.C. (2002). Rac activation induces NADPH oxidase activity in transgenic COSphox cells, and the level of superoxide production is exchange factor-dependent. J. Biol. Chem..

[31] Pick E. (2014). Role of the rho GTPase Rac in the activation of the phagocyte NADPH oxidase: Outsourcing a key task. Small GTPases.

[32] Geyer M., Wittinghofer A. (1997). GEFs, GAPs, GDIs and effectors: Taking a closer (3D) look at the regulation of Ras-related GTP-binding proteins. Curr. Opin. Struct. Biol..

[33] Bos J.L., Rehmann H., Wittinghofer A. (2007). GEFs and GAPs: Critical elements in the control of small G proteins. Cell.

[34] Heo J. (2011). Redox control of GTPases: From molecular mechanisms to functional significance in health and disease. Antioxid. Redox Signal..

[35] Sigal N., Gorzalczany Y., Sarfstein R., Weinbaum C., Zheng Y., Pick E. (2003). The guanine nucleotide exchange factor trio activates the phagocyte NADPH oxidase in the absence of GDP to GTP exchange on Rac. “The emperor's nw clothes”. J. Biol. Chem..

[36] Heo J., Thapar R., Campbell S.L. (2005). Recognition and activation of Rho GTPases by Vav1 and Vav2 guanine nucleotide exchange factors. Biochemistry.

[37] Chhatriwala M.K., Betts L., Worthylake D.K., Sondek J. (2007). The DH and PH domains of Trio coordinately engage Rho GTPases for their efficient activation. J. Mol. Biol..

[38] Heo J., Campbell S.L. (2005). Mechanism of redox-mediated guanine nucleotide exchange on redox-active rho GTPases. J. Biol. Chem..

[39] Heo J., Raines K.W., Mocanu V., Campbell S.L. (2006). Redox regulation of RhoA. Biochemistry.

[40] Heo J., Wey M., Hong I. (2011). Insight into the 6-thiopurine-mediated termination of the invasive motility of tumor cells derived from inflammatory breast cancer. Biochemistry.

[41] Lamarche N., Hall A. (1994). GAPs for rho-related GTPases. Trends Genet..

[42] Negishi M., Katoh H. (2006). [GEFs and gaps of rho family GTPases]. Tanpakushitsu Kakusan Koso.

[43] Kutys M.L., Yamada K.M. (2015). Rho GEFs and GAPs: Emerging integrators of extracellular matrix signaling. Small GTPases.

[44] Peveri P., Heyworth P.G., Curnutte J.T. (1992). Absolute requirement for GTP in activation of human neutrophil NADPH oxidase in a cell-free system: Role of ATP in regenerating GTP. Proc. Natl. Acad. Sci. U. S. A..

[45] Zhen L., King A.A., Xiao Y., Chanock S.J., Orkin S.H., Dinauer M.C. (1993). Gene targeting of X chromosome-linked chronic granulomatous disease locus in a human myeloid leukemia cell line and rescue by expression of recombinant gp91phox. Proc. Natl. Acad. Sci. U. S. A..

[46] Zielonka J., Cheng G., Zielonka M., Ganesh T., Sun A., Joseph J., Michalski R., O'Brien W.J., Lambeth J.D., Kalyanaraman B. (2014). High-throughput assays for superoxide and hydrogen peroxide: Design of a screening workflow to identify inhibitors of NADPH oxidases. J. Biol. Chem..

[47] Heyworth P.G., Knaus U.G., Xu X., Uhlinger D.J., Conroy L., Bokoch G.M., Curnutte J.T. (1993). Requirement for posttranslational processing of Rac GTP-binding proteins for activation of human neutrophil NADPH oxidase. Mol. Biol. Cell.

[48] Heyworth P.G., Knaus U.G., Settleman J., Curnutte J.T., Bokoch G.M. (1993). Regulation of NADPH oxidase activity by Rac GTPase activating protein(s). Mol. Biol. Cell.

[49] DeLeo F.R., Allen L.A., Apicella M., Nauseef W.M. (1999). NADPH oxidase activation and assembly during phagocytosis. J. Immunol..

[50] Pomerening J.R., Sontag E.D., Ferrell J.E. (2003). Building a cell cycle oscillator: Hysteresis and bistability in the activation of Cdc2. Nat. Cell Biol..

[51] Sha W., Moore J., Chen K., Lassaletta A.D., Yi C.S., Tyson J.J., Sible J.C. (2003). Hysteresis drives cell-cycle transitions in Xenopus laevis egg extracts. Proc. Natl. Acad. Sci. U. S. A..

[52] Doncic A., Skotheim J.M. (2013). Feedforward regulation ensures stability and rapid reversibility of a cellular state. Mol. Cell.

[53] Hoang H.M., Umutesi H.G., Heo J. (2021). Allosteric autoactivation of SOS and its kinetic mechanism. Small GTPases.

[54] Heo J., Halbleib C.M., Ludden P.W. (2001). Redox-dependent activation of CO dehydrogenase from Rhodospirillum rubrum. Proc. Natl. Acad. Sci. U. S. A..

[55] Umutesi H.G., Hoang H.M., Johnson H.E., Nam K., Heo J. (2020). Development of Noonan syndrome by deregulation of allosteric SOS autoactivation. J. Biol. Chem..

[56] Sies H., Jones D.P. (2020). Reactive oxygen species (ROS) as pleiotropic physiological signalling agents. Nat. Rev. Mol. Cell Biol..

[57] Bindoli A., Fukuto J.M., Forman H.J. (2008). Thiol chemistry in peroxidase catalysis and redox signaling. Antioxid. Redox Signal..

[58] Perry J.J., Shin D.S., Getzoff E.D., Tainer J.A. (2010). The structural biochemistry of the superoxide dismutases. Biochim. Biophys. Acta.

[59] Azadmanesh J., Borgstahl G.E.O. (2018). A review of the catalytic mechanism of human manganese superoxide dismutase. Antioxidants (Basel).

[60] Kwong C.H., Adams A.G., Leto T.L. (1995). Characterization of the effector-specifying domain of Rac involved in NADPH oxidase activation. J. Biol. Chem..

[61] Hawkins B.J., Madesh M., Kirkpatrick C.J., Fisher A.B. (2007). Superoxide flux in endothelial cells via the chloride channel-3 mediates intracellular signaling. Mol. Biol. Cell.

[62] Mumbengegwi D.R., Li Q., Li C., Bear C.E., Engelhardt J.F. (2008). Evidence for a superoxide permeability pathway in endosomal membranes. Mol. Cell. Biol..

[63] Fisher A.B. (2009). Redox signaling across cell membranes. Antioxid. Redox Signal..

[64] Parvez S., Long M.J.C., Poganik J.R., Aye Y. (2018). Redox signaling by reactive electrophiles and oxidants. Chem. Rev..

[65] Bertagnolo V., Grassilli S., Bavelloni A., Brugnoli F., Piazzi M., Candiano G., Petretto A., Benedusi M., Capitani S. (2008). Vav1 modulates protein expression during ATRA-induced maturation of APL-derived promyelocytes: A proteomic-based analysis. J. Proteome Res..

[66] Tasseff R., Jensen H.A., Congleton J., Dai D., Rogers K.V., Sagar A., Bunaciu R.P., Yen A., Varner J.D. (2017). An effective model of the retinoic acid induced HL-60 differentiation program. Sci. Rep..

[67] Kurganov B.I., Dorozhko A.K., Kagan Z.S., Yakovlev V.A. (1976). The theoretical analysis of kinetic behaviour of “hysteretic” allosteric enzymes. I. The kinetic manifestations of slow conformational change of an oligomeric enzyme in the Monod, Wyman and Changeux model. J. Theor. Biol..

[68] Frieden C. (1979). Slow transitions and hysteretic behavior in enzymes. Annu. Rev. Biochem..

[69] Neet K.E. (1980). Cooperativity in enzyme function: Equilibrium and kinetic aspects. Methods Enzymol..

[70] Qian H. (2008). Cooperativity and specificity in enzyme kinetics: A single-molecule time-based perspective. Biophys. J..

[71] Abo A., Boyhan A., West I., Thrasher A.J., Segal A.W. (1992). Reconstitution of neutrophil NADPH oxidase activity in the cell-free system by four components: p67-phox, p47-phox, p21rac1, and cytochrome b-245. J. Biol. Chem..

[72] Cross A.R., Erickson R.W., Ellis B.A., Curnutte J.T. (1999). Spontaneous activation of NADPH oxidase in a cell-free system: Unexpected multiple effects of magnesium ion concentrations. Biochem. J..

[73] Shin J.Y., Wey M., Umutesi H.G., Sun X., Simecka J., Heo J. (2016). Thiopurine prodrugs mediate immunosuppressive effects by interfering with Rac1 protein function. J. Biol. Chem..

[74] Dahlgren C., Karlsson A. (1999). Respiratory burst in human neutrophils. J. Immunol. Methods.

[75] Lenzen C., Cool R.H., Wittinghofer A. (1995). Analysis of intrinsic and CDC25-stimulated guanine nucleotide exchange of p21ras-nucleotide complexes by fluorescence measurements. Methods Enzymol..

[76] Gureasko J., Galush W.J., Boykevisch S., Sondermann H., Bar-Sagi D., Groves J.T., Kuriyan J. (2008). Membrane-dependent signal integration by the Ras activator son of sevenless. Nat. Struct. Mol. Biol..

[77] Wey M., Lee J., Jeong S.S., Kim J., Heo J. (2013). Kinetic mechanisms of mutation-dependent Harvey Ras activation and their relevance for the development of Costello syndrome. Biochemistry.

[78] Wey M., Lee J., Kim H.S., Jeong S.S., Kim J., Heo J. (2016). Kinetic mechanism of formation of hyperactive embryonic Ras in cells. Biochemistry.

[79] Teufelhofer O., Weiss R.M., Parzefall W., Schulte-Hermann R., Micksche M., Berger W., Elbling L. (2003). Promyelocytic HL60 cells express NADPH oxidase and are excellent targets in a rapid spectrophotometric microplate assay for extracellular superoxide. Toxicol. Sci..

[80] Seitz P.M., Cooper R., Gatto G.J., Ramon F., Sweitzer T.D., Johns D.G., Davenport E.A., Ames R.S., Kallal L.A. (2010). Development of a high-throughput cell-based assay for superoxide production in HL-60 cells. J. Biomol. Screen..

