# Supplementary material for: Rac-dependent feedforward autoactivation of NOX2 leads to oxidative burst
Source: J Biol Chem. 2021 Jul 20;297(2):100982. doi: 10.1016/j.jbc.2021.100982 (PMC8353492; doi:10.1016/j.jbc.2021.100982)
Supplement: Supporting information [file mmc1.pdf]

Supporting Information

Half-life of superoxide under various conditions. Studies have shown that the half-life of O_2^- can span many orders of magnitude (milliseconds to hours) primarily depending on its concentration, the concentration and identity of its reaction partners, and numerous environmental factors such as pH (1,2). Localization and compartmentalization of key enzymes and their associated reactants and products is one mechanism that nature has used for this regulation. The half-life and subsequent diffusion distance of O_2^- varies markedly depending on cellular context. This is an unsurprising observation given that O_2^- is involved in a variety of diverse signaling pathways and even plays a role as an intermediate species in the generation of high concentrations of reactive species for the destruction of pathogens.

O_2^- decay routes. Each reaction contributing to the decrease of O_2^- over time can be considered a 'decay route'. Each decay route, such as the spontaneous disproportionation of O_2^-, enzyme catalyzed reactions involving O_2^-, or reactions between O_2^- and other species, contributes to the overall O_2^- decay rate. A few O_2^- decay routes are briefly discussed below.

O_2^- spontaneously disproportionates to hydrogen peroxide. The rate of spontaneous O_2^- disproportionation strongly depends on pH due to the equilibrium between O_2^- and its conjugate acid, hydroperoxyl radical (HO_2), which has a pK_a around 4.8 (3). At the pK_a, the observed second-order rate constant (k_{obs}) for the spontaneous disproportionation of O_2^- is maximal, $k_{obs} > 10^7$ $M^{-1}s^{-1}$; accordingly, the rate of spontaneous O_2^- disproportionation is maximal. For each pH unit above the pK_a, the rate is reduced by a factor of 10 due to increased anion repulsion (4). Bielski and colleagues have shown that at pH > 6, k_{obs} for the spontaneous disproportionation of O_2^- linearly depends on pH (Eq. 1).

$$k_{obs} = 6 \times 10^{12} [H^+] (M^{-1}s^{-1}) \qquad \text{Eq. 1}$$

Thus, at pH 7.4, k_{obs} for spontaneous disproportionation is 2.4×10^5 $M^{-1}s^{-1}$.

Enzyme catalyzed reactions and the reactions between O_2^- and other species are major determinants of O_2^- decay. SOD catalyzed O_2^- disproportionation is expected to be the main route of O_2^- decay in the cellular environment as SOD increases the rate of spontaneous disproportionation dramatically and is a ubiquitously present enzyme. O_2^- also reacts very quickly with nitric oxide (NO), to form the highly reactive peroxynitrite anion ($ONOO^-$). Both reactions are nearly diffusion controlled with values of the true second-order rate constant (k) > 10^9 $M^{-1}s^{-1}$ (Table S1). O_2^- also reacts very quickly with transition metals such as those found in [Fe_4-S_4] clusters of some proteins; however, given that these reactive centers are generally sequestered within proteins, this is not expected to contribute significantly to the decay rate of O_2^-. The most prominent O_2^- decay routes can be assessed by comparing the concentration of reactants in the vicinity of O_2^- and the rate constants of each of the respective reactions. Various reactions contributing to O_2^- decay and their associated rate constants are listed in Table S1.

Table S1. *Biologically relevant reactions contributing to O_2^- decay.*

Reactions	Rate constants[a] (M^{-1} s^{-1})	pH	Location[b]	Sources	Ref.[c]
Uncatalyzed O_2^- disproportionation					
$HO_2 + HO_2 \rightarrow H_2O_2 + O_2$	$k = (8.3 \pm 0.7) \times 10^5$				(3)
$HO_2 + O_2^- + H_2O \rightarrow H_2O_2 + O_2 + OH^-$	$k = (9.7 \pm 0.6) \times 10^7$				(3)
$O_2^- + O_2^- + 2H_2O \rightarrow H_2O_2 + O_2 + 2OH^-$	$k < 100$				(5)
SOD catalyzed O_2^- disproportionation					

Extracellular SOD (Cu, Zn)	$k = 1 \times 10^9$	9.5	PM, S	Human	(6)
SOD (Cu, Zn)	$k_{obs} = 1.5 \times 10^9$	9.5	C, M, N	Human	(5)
	$k_{obs} = 3 \times 10^9$	9.5		Bovine	(5)
	$k = {\sim}2 \times 10^9$	5.0-9.5		Bovine	(7)
SOD (Mn)	$k_{obs} = 4 \times 10^8$	9.5	M	Bovine	(5)
	$k_{obs} = (1.5 \pm 0.15) \times 10^9$	7.9		*E. coli*	(8)
$O_2^- + NO \rightarrow ONOO^-$	$k = (6.7 \pm 0.9) \times 10^9$	5.6-12.5			(9)
	$k = (4.3 \pm 0.5) \times 10^9$	6.1-10.0			(10)
$HO_2 + NO \rightarrow ONOOH$	$k = (3.2 \pm 0.3) \times 10^9$	3.3			(10)
Aconitase	$k = {\sim}3 \times 10^7$	8.0	C, M	*E. coli* & human cytosolic aconitase	(11)
	$k = {\sim}8 \times 10^6$	8.0		Porcine mitochondrial aconitase	(11)
Cytochrome *c* (Cyt *c*) $O_2^- + \text{Cyt } c\ (Fe^{3+}) \rightarrow O_2 + \text{Cyt } c\ (Fe^{2+})$	$k = (3.0 \pm 0.4) \times 10^5$	7.45-9.2	M		(12)
Ascorbic acid (AH_2) $HO_2/O_2^- + AH_2/AH^- \rightarrow A^- + H_2O_2$	$k_{obs} = 2.7 \times 10^5$	7.4			(3)
Glutathione (GSH) $O_2^- + GSH + H^+ \rightarrow GS + H_2O_2$	$k = 6.7 \times 10^5$	7.8			(13)

[a]The kinetic parameter k indicates a true second-order rate constant, while k_{obs} reflects an observed second-order rate constant. Note that k is independent of the system composition and time, whereas k_{obs} is reported for reactions under a set of specified conditions and depends on system composition. [b]The cellular location of various enzymes is indicated. [c]Reference cited. C, cytosol; N, nucleus; M, mitochondria; S, secreted; PM, plasma membrane.

Decay rate of superoxide under various conditions. The most prominent O_2^- decay routes need to be considered to estimate the decay rate of O_2^-, and the subsequent half-life of O_2^-. The decay rate ($df(t)/dt$) of O_2^- can be described by the following equation:

$$\frac{d\,f(t)}{dt} = -\left(k_1 f(t)^2 + k_2[\text{SOD}]f(t) + \cdots + \sum k_i\,[\text{S}]f(t)\right) \qquad \text{Eq. 2}$$

where $f(t)$ is the O_2^- concentration at time t, k_1 is the second-order rate constant for spontaneous O_2^- disproportionation, k_2 is the second-order rate constant for SOD-catalyzed O_2^- disproportionation, bracketed terms represent concentrations of the reaction partners, and $\sum k_i\,[\text{S}]f(t)$ is the sum of other O_2^- decay routes where k_i represents the respective rate constant for reactions with a species, S. Note that when solving this equation, the concentration of O_2^- reaction partners (e.g., SOD) and the pH are assumed to remain constant. It should be emphasized that the rate of O_2^- formation is not accounted for here; this equation describes the decay of a bolus of O_2^- in a homogenous solution.

Estimating the diffusion distance of O_2^-. Determining the diffusion distance of O_2^- in a cellular environment using Fick's second law of diffusion is a difficult endeavor. This is in part due to the complicated time dependence of O_2^- concentration that results from its numerous routes of formation and decay, the dependence of diffusion on the concentration gradient, the geometry and inhomogeneity of the system, other forces acting on O_2^- (e.g., anion repulsion), among other factors. However, if O_2^- is

considered a point source in a spherically symmetrical environment, its diffusion distance can be approximated using Eq. 3 (14,15).

$$\chi = 2.26(D\tau)^{1/2} \qquad \text{Eq. 3}$$

where χ is the average path length in solution, D is the diffusion coefficient for O_2^-, and τ is the half-life of O_2^-. The estimated diffusion distance using this equation represents the radius of a sphere; in other words, the diffusion of O_2^- in any direction is considered equally probable (16). In cases where the diffusion of O_2^- is partially bounded, for instance if O_2^- is produced near a section of membrane that is less permeable to O_2^-, the diffusion distance outward from this barrier is expected to be greater. In aqueous solution, D for O_2^- is $\sim 1.5\times10^{-9} m^2 s^{-1}$ (17). However, it has been shown that the translational mobility of small solutes is reduced by a factor of ~4 in the cell cytoplasm relative to aqueous solutions (18).

The estimated half-life and diffusion distance for O_2^- determined under various conditions is shown in Table S2. For simplicity, only spontaneous O_2^- disproportionation and SOD-catalyzed O_2^- disproportionation were accounted for in our calculations. From these results, it is apparent that SOD has the most dramatic effect on O_2^- at low concentrations of O_2^-. This is because spontaneous O_2^- decay has second-order dependence on O_2^- concentration, while SOD-catalyzed O_2^- disproportionation has only first-order dependence on O_2^- concentration. Furthermore, we see that at low concentrations of SOD and O_2^-, O_2^- can persist for surprisingly long periods of time which may enable it to act in an autocrine or paracrine fashion. It is feasible that extracellular O_2^- released from one cell can diffuse to adjacent cells where it may be imported into the cell (e.g., through ClC-3 channels) to influence the process of NOX2 autoactivation, or perhaps play signaling roles initiated at the cell surface. It should be noted that this analysis only yields an estimate of O_2^- diffusion distance in a hypothetical, idealized space. In a more detailed analysis, the number of assumptions/simplifications used should be reduced; the contribution of anion channels to O_2^- import and the microenvironment near the plasma membrane should also be considered. For instance, the plasma membrane attracts positive counterions, including H^+, which may decrease the pH in its vicinity (19). Although the decrease in pH may lead to increased O_2^- decay, the increased generation of HO_2, which diffuses through the plasma membrane much more readily than O_2^- (20), may represent a potentially important mechanism for O_2^- import in cells, in addition to O_2^- import through ClC-3 channels (discussed in main text). In conclusion, although these calculations may suffer from oversimplification, claims that O_2^- may function as a paracrine or autocrine signaling molecule appear reasonable.

Table S2. *Estimated half-life and diffusion distance of O_2^- under various conditions.*

Concentration (M)		Half-life (s)	Diffusion distance (μm)
O_2^-	SOD[a]		
10^{-9}	-	9.9×10^3	7.1×10^3
	10^{-9}	7.1×10^{-1}	6.0×10^1
	10^{-6}	6.8×10^{-4}	1.9
10^{-8}	-	9.9×10^2	2.3×10^3
	10^{-9}	7.1×10^{-1}	6.0×10^1
	10^{-6}	6.9×10^{-4}	1.9
10^{-7}	-	9.9×10^1	712
	10^{-9}	6.8×10^{-1}	59
	10^{-6}	6.8×10^{-4}	1.9
10^{-6}	-	1.0×10^1	2.3×10^2
	10^{-9}	6.5×10^{-1}	58

	10^{-6}	6.9×10^{-3}	1.9
10^{-5}	-	1.0	71
	10^{-9}	4.1×10^{-1}	46
	10^{-6}	6.9×10^{-4}	1.9
10^{-4}	-	1.0×10^{-1}	23
	10^{-9}	8.7×10^{-2}	21
	10^{-6}	6.9×10^{-4}	1.9
10^{-3}	-	1.0×10^{-2}	7.1
	10^{-9}	9.9×10^{-3}	7.1
	10^{-6}	6.5×10^{-4}	1.8

[a]We used $k_1 = 10^5$ M^{-1} s^{-1} and $k_2 = 10^9$ M^{-1} s^{-1} corresponding to uncatalyzed superoxide decay and extracellular SOD (Cu, Zn) catalyzed decay, respectfully. D of O_2^- was set to be 10^{-9}m^{2}s^{-1}.

Kinetic model of the Rac-dependent NOX2 autoactivation. Of the several functions of the kinetic model that describes the hysteresis of natural processes over time, the time-dependent logistic function (Eq. 4) fits best for NOX2 autoactivation. This is because the time-dependent logistic function accounts for the kinetic hysteresis of the action of the feedback loop between the wt Rac and NOX2 activations over time.

$$\gamma_t^a = \frac{1}{1 + \frac{[\mathrm{NOX2}_{t=0}^i]}{[\mathrm{NOX2}_{t=0}^a]} e^{-k_{\mathrm{auto}} t}} = \frac{1}{1 + \frac{[\mathrm{Rac}_{t=0}^i]}{[\mathrm{Rac}_{t=0}^a]} e^{-k_{\mathrm{auto}} t}} \qquad \text{Eq. 4}$$

γ_t^a represents the fraction of active NOX2 at given time *t*. The parameter k_{auto} is a rate constant of NOX2 autoactivation. $[\mathrm{NOX2}_{t=0}^i]$ and $[\mathrm{NOX2}_{t=0}^a]$ are, respectively, the fractions of inactive and active NOX2 at time 0. $[\mathrm{Rac}_{t=0}^i]$ and $[\mathrm{Rac}_{t=0}^a]$ are, respectively, the fractions of inactive and active NOX2 at time 0. NOX2 autoactivation is a reflection of the wt Rac autoactivation. Therefore, the equation with NOX2 (left column of Eq. 4) can essentially be substituted for the equation with wt Rac (right column of Eq. 4). Note that, except for different modifiers, Eq. 4 is essentially the same equation as for the autocatalysis of carbon monoxide dehydrogenase (CODH) and Son of Sevenless (SOS) autoactivation. This is because, as with NOX2 autoactivation, the time-dependent logistic function is the best fit for the kinetics of CODH autocatalysis (21) as well as SOS autoactivation (22,23).

Autoactivation-specific kinetic parameters of the NOX2 activation. Recognition of the NOX2 autoactivation feature, in combination with Eq. 4, leads us to determine the NOX2 autoactivation specific-kinetic parameters that describe the previously unknown mechanistic features of the NOX2 activation.

<u>Autoactivation rate constant</u>. The kinetic parameter, k_{auto}, in Eq. 4 is an autoactivation specific-rate constant. This parameter reflects the speed of the NOX2 autoactivation. The value interpretation of this autoactivation kinetic parameter should be the opposite of the typical first- or second-order rate constants that describe the speed of the enzyme catalytic actions. This is because a smaller value of the autoactivation specific-rate constant denotes faster autoactivation, whereas a smaller first- or second-order rate constant indicates slower kinetic processes.

The best fits of the hysteretic wt Rac and NOX2 activations over time with Eq. 4 gave the k_{auto} values of the wt Rac and NOX2 autoactivation processes (Figure 5 of the main text, redlines). Intriguingly, the k_{auto} value of wt Rac autoactivation is all-but indistinguishable from that of NOX2 autoactivation (Figure 5 of the main text, detailed k_{auto} value analysis is in the next section). As noted above, the hysteretic wt Rac

and NOX2 activations over time, respectively, reflect the mechanistic wt Rac autoactivation that confers functional NOX2 autoactivation. Therefore, the similarity of k_{auto} values suggests that, kinetically, whenever wt Rac is autoactivated, it immediately causes functional NOX2 autoactivation. This is not too surprising because NOX2 activity tightly dovetails with wt Rac activity.

Threshold of the autoactivation initiator. The necessity of a reaction trigger, initiator, or primer is one of the unique features of cyclic reaction processes, including enzyme autoactivation, combustion engine operations, and the burning process of fire. In the case of NOX2 autoactivation, either active wt Rac or the active NOX2 complex can serve as the trigger. This is because both function to produce the O_2^- that targets wt Rac to activate wt Rac, initiating the NOX2 autoactivation cycle. Once this cyclic process is initiated, it generates more reaction triggers that amplify and thus automate the cyclic reaction process.

In theory, any fraction of the reaction trigger, no matter how small, can initiate the autoactivation cycle. However, in the case of many enzymatic systems, including NOX2, whenever a reaction trigger is introduced into the cyclic reaction system to initiate its autoactivation, a fraction of the reaction trigger can intrinsically decay over time. Therefore, in reality, initiation of enzyme autoactivation requires a fraction of the reaction trigger large enough to overcome its eventual decay. Likewise, in the case of the NOX2 system, a sufficient fraction of active wt Rac — the reaction trigger — is necessary to initiate NOX2 autoactivation. Within the NOX2 system, the minimally active wt Rac fraction capable of initiating NOX2 autoactivation is defined as the threshold for initiation of NOX2 autoactivation. This threshold for the reaction trigger is expressed as a molar proportion (mole %) of the active wt Rac with inactive wt Rac ($\frac{[\mathrm{Rac}^a_{t=0}]}{[\mathrm{Rac}^i_{t=0}]}$), which is formulated inversely as a denominator in Eq. 4. When combined, in addition to the k_{auto} value, the threshold value of the wt Rac trigger also is a key kinetic parameter that describes the features of NOX2 autoactivation. Accordingly, determination of the threshold value of the wt Rac trigger is essential in deciphering the kinetic mechanism of regulation of NOX2 function.

Treatments and separations of the cell-free NOX2 system components. The rhodamine-, kinase, GDP, and GTP-treated cell-free assay protein samples were further purified by using a size-exclusion FPLC approach.

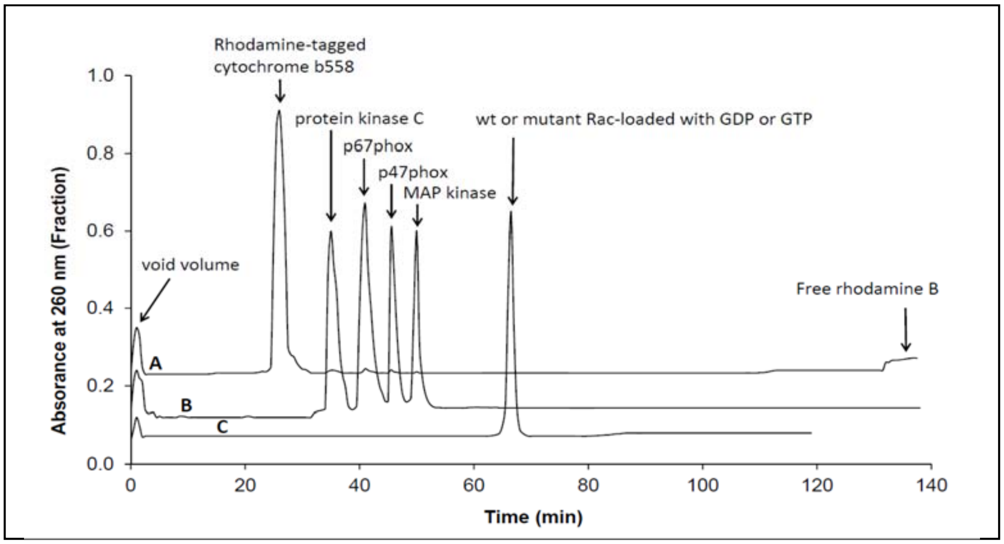

Figure S1. *Size exclusion chromatography of cell-free system components*. BioLogic LP system (Bio-Rad Laboratories) equipped with a Superdex 6 column (for sample volume 13 mL) was used for this

analysis. Concentrations of all protein samples of each analysis were adjusted to 50 μM. **A**. Cytochrome b558 was treated with rhodamine as indicated in the Experimental Procedures section. The rhodamine B-treated sample was applied onto the column and then eluted with ammonium acetate buffer (pH 7.0). The flow rate was 0.75 mM/min. **B**. p67 and p47 were treated with MAP kinase and protein kinase C as indicated in the Experimental Procedures section. The sample mixture was then applied onto and eluted from the column as noted above. **C**. Active GTP-loaded or inactive GDP-loaded Rac sample was also applied onto and then eluted from the column as noted above.

References

1. Sheng, Y., Abreu, I. A., Cabelli, D. E., Maroney, M. J., Miller, A. F., Teixeira, M., and Valentine, J. S. (2014) Superoxide dismutases and superoxide reductases. *Chemical Reviews* **114**, 3854-3918
2. Winterbourn, C. C. (2008) Reconciling the chemistry and biology of reactive oxygen species. *Nature Chemical Biology* **4**, 278-286
3. Bielski, B. H. J., Cabelli, D. E., Arudi, R. L., and Ross, A. B. (1985) Reactivity of HO_2/O_2^- Radicals in Aqueous Solution. *Journal of Physical and Chemical Reference Data* **14**, 1041-1100
4. Fridovich, I. (1983) SUPEROXIDE RADICAL: AN ENDOGENOUS TOXICANT. *Annual Review of Pharmacology and Toxicology* **23**, 239-257
5. Marklund, S. (1976) Spectrophotometric study of spontaneous disproportionation of superoxide anion radical and sensitive direct assay for superoxide dismutase. *Journal of Biological Chemistry* **251**, 7504-7507
6. Marklund, S. L. (1982) Human copper-containing superoxide dismutase of high molecular weight. *Proceedings of the National Academy of Sciences of the United States of America* **79**, 7634-7638
7. Forman, H. J., and Fridovich, I. (1973) Superoxide dismutase: A comparison of rate constants. *Archives of Biochemistry and Biophysics* **158**, 396-400
8. Pick, M., Rabani, J., Yost, F., and Fridovich, I. (1974) The Catalytic Mechanism of the Manganese-Containing Superoxide Dismutase of Escherichia coli Studied by Pulse Radiolysis. *Journal of the American Chemical Society* **96**, 7329-7333
9. Huie, R. E., and Padmaja, S. (1993) The reaction of no with superoxide. *Free Radical Research* **18**, 195-199
10. Goldstein, S., and Czapski, G. (1995) The reaction of $NO^•$ with $O_2^{•-}$ and $HO_2^{•-}$: A pulse radiolysis study. *Free Radical Biology and Medicine* **19**, 505-510
11. Hausladen, A., and Fridovich, I. (1994) Superoxide and peroxynitrite inactivate aconitases, but nitric oxide does not. *Journal of Biological Chemistry* **269**, 29405-29408
12. Butler, J., Jayson, G. G., and Swallow, A. J. (1975) The reaction between the superoxide anion radical and cytochrome c. *BBA - Bioenergetics* **408**, 215-222
13. Asada, K., and Kanematsu, S. (1976) Reactivity of Thiols with Superoxide Radicals. *Agricultural and Biological Chemistry* **40**, 1891-1892
14. Saran, M., and Bors, W. (1994) Signalling by $O_2^{-•}$ and $NO^•$: how far can either radical, or any specific reaction product, transmit a message under in vivo conditions? *Chemico-Biological Interactions* **90**, 35-45
15. Roots, R., and Okada, S. (1975) Estimation of life times and diffusion distances of radicals involved in X ray induced DNA strand breaks or killing of mammalian cells. *Radiation Research* **64**, 306-320
16. Lancaster, J. R. (1996) Diffusion of Free Nitric Oxide. in *Methods in Enzymology*. pp 31-50
17. Divišek, J., and Kastening, B. (1975) ELECTROCHEMICAL GENERATION AND REACTIVITY OF THE SUPEROXIDE ION IN AQUEOUS SOLUTIONS. *Journal of Electroanalytical Chemistry* **65**, 603-621
18. Kao, H. P., Abney, J. R., and Verkman, A. S. (1993) Determinants of the Translational Mobility of a Small Solute in Cell Cytoplasm. *The Journal of Cell Biology* **120**, 175-184

19. Freeman, B. A., and Crapo, J. D. (1982) Biology of disease Free radicals and tissue injury. *Laboratory Investigation* **47**, 412-426
20. Möller, M. N., Cuevasanta, E., Orrico, F., Lopez, A. C., Thomson, L., and Denicola, A. (2019) Diffusion and transport of reactive species across cell membranes. pp 3-19
21. Heo, J., Halbleib, C. M., and Ludden, P. W. (2001) Redox-dependent activation of CO dehydrogenase from Rhodospirillum rubrum. *Proc Natl Acad Sci U S A* **98**, 7690-7693
22. Hoang, H. M., Umutesi, H. G., and Heo, J. (2019) Allosteric autoactivation of SOS and its kinetic mechanism. *Small GTPases*, 1-16
23. Umutesi, H. G., Hoang, H. M., Johnson, H. E., Nam, K., and Heo, J. (2020) Development of Noonan syndrome by deregulation of allosteric SOS autoactivation. *The Journal of biological chemistry* **295**, 13651-13663
